# Automated Analysis of Vertebral Body Surface Roughness for Adult Age Estimation: Ellipse Fitting and Machine-Learning Approach

**DOI:** 10.3390/diagnostics15141794

**Published:** 2025-07-16

**Authors:** Erhan Kartal, Yasin Etli

**Affiliations:** Department of Forensic Medicine, Van Yüzüncü Yıl University, Van 65090, Turkey; yasinetli@yyu.edu.tr

**Keywords:** age estimation, vertebral surface roughness, ellipse fitting, computed tomography, machine learning, forensic radiology

## Abstract

**Background/Objectives:** Vertebral degenerative features are promising but often subjectively scored indicators for adult age estimation. We evaluated an objective surface roughness metric, the “average distance to the fitted ellipse” score (DS), calculated automatically for every vertebra from C7 to S1 on routine CT images. **Methods:** CT scans of 176 adults (94 males, 82 females; 21–94 years) were retrospectively analyzed. For each vertebra, the mean orthogonal deviation of the anterior superior endplate from an ideal ellipse was extracted. Sex-specific multiple linear regression served as a baseline; support vector regression (SVR), random forest (RF), k-nearest neighbors (k-NN), and Gaussian naïve-Bayes pseudo-regressor (GNB-R) were tuned with 10-fold cross-validation and evaluated on a 20% hold-out set. Performance was quantified with the standard error of the estimate (SEE). **Results:** DS values correlated moderately to strongly with age (peak r = 0.60 at L3–L5). Linear regression explained 40% (males) and 47% (females) of age variance (SEE ≈ 11–12 years). Non-parametric learners improved precision: RF achieved an SEE of 8.49 years in males (R^2^ = 0.47), whereas k-NN attained 10.8 years (R^2^ = 0.45) in women. **Conclusions:** Automated analysis of vertebral cortical roughness provides a transparent, observer-independent means of estimating adult age with accuracy approaching that of more complex deep learning pipelines. Streamlining image preparation and validating the approach across diverse populations are the next steps toward forensic adoption.

## 1. Introduction

Age estimation—alongside sex and stature assessment—is a critical step in establishing the identity of skeletal remains in forensic anthropological investigations [1]. Beyond this context, determining skeletal age is also essential in clinical forensic medicine [2], forensic radiology [3], and bio-archaeological research [1,4]. Accurate adult age attribution is far more than a taxonomic exercise: it underpins victim identification, informs medico-legal responsibility, and shapes demographic reconstructions. Casework experience shows that even a ±5- to 10-year misclassification can shift an unidentified skeleton into the wrong search stratum of missing persons databases or obscure a match when only partial antemortem information is available [1,5]. In the living, age estimates guide judicial decisions on criminal liability, asylum status and elite sport eligibility. Mansour et al. documented how erroneous age opinions in Hamburg led to both unwarranted detention and premature release of minors [2]. Radiology teams are therefore exploring automated pipelines that can infer age from routine imaging (e.g., chest radiographs [3]) or integrate multimodal deep learning forecasts [4] to tighten precision. However, classical morphoscopic methods remain limited by observer error and population specificity, prompting continuous refinement—such as transition analysis [6] and 3-D virtual validation of long-standing schemes in new populations [7]. Parallel biochemical, genetic, and epigenetic assays are now entering forensic practice, but they too report error bands that must be weighed against skeletal approaches [8]. Recent work even links inaccurate age estimation to biased interpretations of frailty and cause of death in paleopathological samples, illustrating how methodological drift can distort broader epidemiological narratives [9]. Collectively, these lines of evidence underscore the idea that improving the accuracy and transparency of adult age-estimation techniques—such as the surface roughness metric proposed here—is not merely an academic refinement but a prerequisite for ethical and legally defensible forensic science [10].

Although chronological age in subadults can be approximated with acceptable error margins by monitoring skeletal development [1,5,6], age estimation in adult populations remains a major practical challenge [1,10]. Adult methods exploit bone degeneration—so-called “wear-and-tear” changes—but the expression of these traits shows marked variability both among individuals and across populations [10]. Such heterogeneity stems from inter-individual and inter-population differences in genetic [7,8], environmental [7], and epigenetic influences [8], as well as variability in disease burden and overall health status [9], habitual physical activity [11], and body size [7]. Consequently, there is a substantial need for reliable alternative approaches to adult age estimation, and efforts to develop and standardize such methods are still ongoing [12,13].

Contemporary adult age-estimation protocols typically rely on skeletal indicators such as cranial suture closure, dental wear, auricular surface of the ilium, degenerative changes in the pubic symphysis, and the metamorphic stages observed at the sternal ends of the ribs [14]. Because current techniques do not yet yield sufficiently reliable results, the vertebrae have recently gained attention as one of the additional skeletal elements whose potential utility for age estimation is under active investigation [13,15,16,17,18,19,20,21,22,23,24,25,26,27,28,29,30,31,32,33,34]. Among vertebral indicators evaluated for adult age estimation, osteophyte formation has emerged as the most prominent feature [13,15,17,19,21,24,25,26,28,31]. Osteophytes are known to develop with advancing age at the margins of the intervertebral discs throughout the vertebral column. Additional age-related changes include deformation and increasing concavity of the vertebral bodies, as well as progressive ossification of the ligaments between adjacent vertebrae [17]. Additionally, cumulative micro-trauma, bone loss, and heightened osteoclastic activity with advancing age result in cortical thinning, increased porosity, and irregular indentations and protrusions. Collectively, these morphological alterations enhance the roughness of the bone surface [11].

Many techniques devised to characterize these vertebral surface changes still depend on subjective scoring systems [13,15,17,19,21,22,25,31]. Although numerous image-processing approaches could, in principle, provide an objective quantification of “roughness,” our literature survey revealed only a few studies that have addressed this issue directly [26,32,34]. One investigation explored the applicability of the convex-hull-derived volume ratio—a proxy metric for surface irregularity—and reported promising accuracy for vertebral age estimation [26]. In a separate study by the same team, the L4 vertebral body was smoothed using ITK-SNAP’s smoothing feature, and the maximum Hausdorff distance was then computed in MeshLab. This vertebral body roughness metric was subsequently used for age estimation [32]. In another study conducted by our group—designed as a preliminary investigation for the present project—we applied the newly proposed method to quantify roughness of the sacral base for age estimation and similarly obtained encouraging results [34].

In this cross-sectional study, we analyzed CT images of all 19 vertebral bodies from C7 to S1 in 176 living adults (21–94 years, 94 males and 82 females) using our previously introduced ellipse-fitting roughness algorithm. We hypothesized that vertebral cortical roughness would correlate positively with chronological age, providing a reliable indicator for adult skeletal age estimation. Furthermore, we aimed to show that this automated metric offers a repeatable, objective alternative to existing subjective scoring systems and can be readily adopted in future research and forensic practice.

The remainder of this article is structured as follows: Section 2 (Materials and Methods) describes the study cohort, CT acquisition parameters, the DS extraction workflow (image preparation, contour detection, ellipse fitting), and the statistical learning framework—each in its own numbered sub-section. Section 3 (Results) reports the descriptive statistics, sex-specific correlation patterns, and the comparative performance of linear, LASSO, and four machine learning regressors. Section 4 (Discussion) interprets the findings under nine thematic sub-headings: main results, comparison with conventional scores, contrast with deep learning pipelines, population/sex patterns, model diagnostics, practical applications, ethical–legal issues, biological rationale, and taphonomic considerations (Section 4.1, Section 4.2, Section 4.3, Section 4.4, Section 4.5, Section 4.6, Section 4.7, Section 4.8 and Section 4.9). Section 5 (Limitations) summarizes methodological constraints and avenues for improvement, while Section 6 (Conclusions) distills the key take-home messages and outlines future work. Appendix figures, full diagnostic plots, and the open-access dataset/code are provided in the Appendix A and Zenodo repository referenced at the end of the manuscript.

## 2. Materials and Methods

### 2.1. Sample Size and Demographics

**Figure A1:**
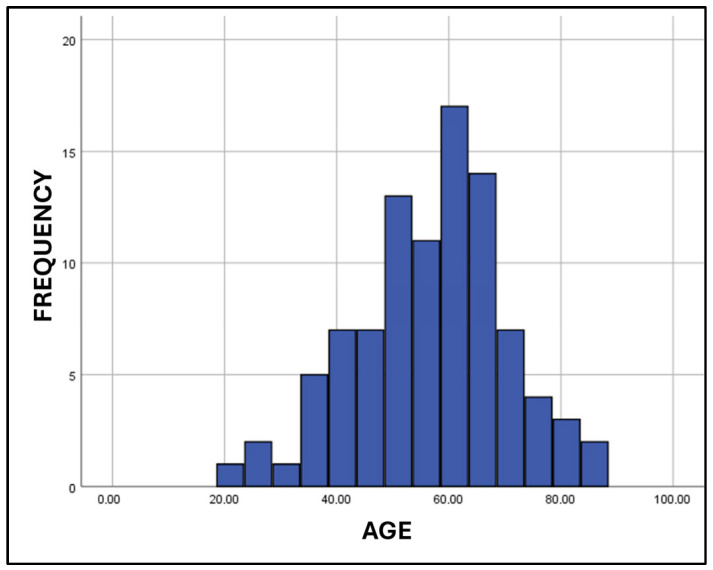
Histogram showing the distribution of ages of male cases.

**Figure A2:**
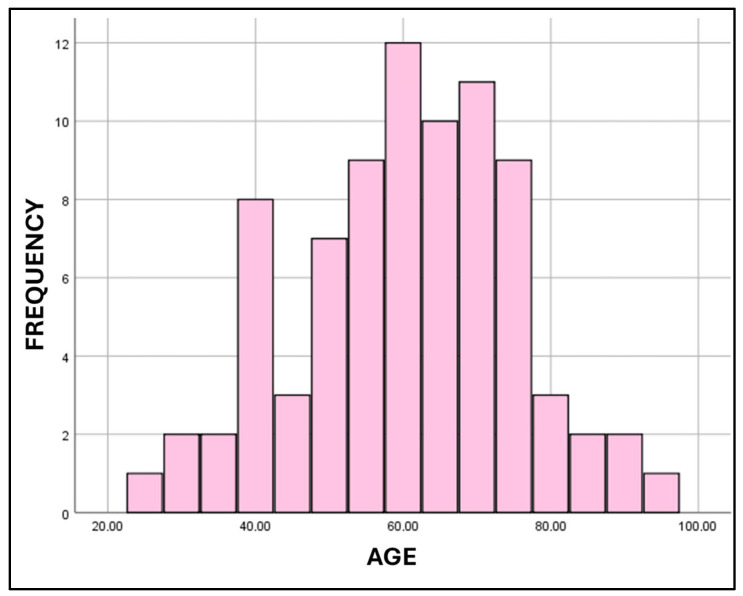
Histogram showing the distribution of ages of female cases.

**Figure A3:**
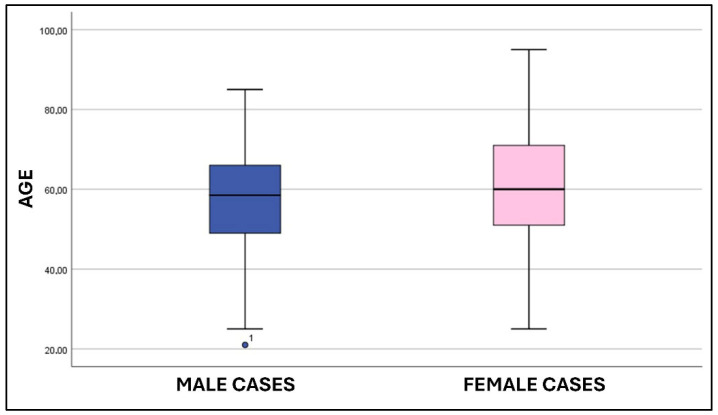
Box-plot graph showing the comparative age distribution of male and female cases.

A total of 176 adult CT examinations (94 males, 82 females) were included. Chronological age ranged from 21 to 94 years (Mean ± SD = 58.5 ± 14.0 y; males 57.0 ± 13.2 y, females 60.2 ± 14.7 y). Detailed age/sex distribution is provided in Figure A1, Figure A2 and Figure A3 and Table A1.

### 2.2. Case Selection

This retrospective study was conducted at Van Yüzüncü Yıl University Dursun Odabaş Medical Center. Spine CT examinations performed between 1 January 2020 and 31 October 2023 were retrieved from the hospital picture-archiving and communication system. Adults aged ≥ 21 years who had a single thoracic–lumbar scan encompassing vertebrae C7 through S1 on the index date were screened for inclusion.

Inclusion criteria: (1) age ≥ 21 years; (2) CT series demonstrating the entire vertebral column from C7 to S1.

Exclusion criteria: (1) metabolic, endocrine, or neoplastic disorders—or prior fracture, surgery, or instrumentation—expected to alter bone density or morphology; (2) congenital anomalies affecting any vertebra between C7 and S1; (3) motion, metallic, or other artefacts obscuring cortical detail; (4) CT studies that omitted one or more of the target vertebrae.

Only individuals ≥ 21 years were eligible, because the vertebral ring-apophyses (secondary ossification centers) normally fuse with the vertebral body by 18–20 y in females and 19–23 y in males; after this epiphyseal closure, cortical topography reflects degenerative rather than growth-related remodeling, which is the biological signal our DS metric is intended to capture [15,30,35].

Electronic health record data were reviewed, under institutional ethics approval, to verify that no candidate met any exclusion criterion. To achieve random case selection, CT examinations were screened in reverse chronological order beginning on 31 October 2023 and working backwards until equal numbers of male and female adults (≥21 years), roughly equally distributed in age groups, with a single thoracic–lumbar series covering C7–S1 were found. The target of 100 men and 100 women was reached with scans dated 15 August 2022 or later. These 200 cases were then scrutinized for the predefined exclusion criteria, resulting in the removal of 18 women and 6 men. The final analytic cohort therefore comprised 176 individuals—82 women and 94 men.

### 2.3. Radiologic Imaging

In this study, surface roughness was quantified by scaling the boundary coordinates of the anterior half of the vertebral corpus superior end plate, isolated from 2D tomographic slices. The surface roughness metric is derived from the antero-superior end-plate cortex because:

(i) This surface bears the highest compressive and shear loads during standing and flexion;
(ii) Cortical porosity and scalloping on this face have been shown to increase monotonically with age [15,16];
(iii) The outline of the intact anterior half approximates an ellipse in young adults, providing a mathematically convenient reference shape. The posterior half was purposefully excluded to avoid pedicle interference and because its load path is dominated by trabecular rather than cortical bone. By fitting a least-squares ellipse to the intact anterior rim and computing the mean orthogonal distance of every boundary node to that ellipse, we obtain a single, observer-independent roughness index that quantifies cumulative cortical remodeling.

Thoracic and lumbar CT scans were obtained in helical mode on either a 16-slice SOMATOM Emotion^®^ (software VA40A, Siemens Healthineers, Erlangen, Germany) or a 64-slice SOMATOM Sensation^®^ multidetector scanner (software VB10B, Siemens Healthineers, Erlangen, Germany) under the following parameters: 120 kVp, 120–150 mAs, collimation 16 × 1.5 mm, pitch = 1.1, and rotation time = 0.5 s. Raw data were acquired at 0.6–1 mm slice thickness and reconstructed as 1 mm axial and 2 mm sagittal/coronal multiplanar reformations using bone (B70) and soft-tissue (B30) kernels. The resulting DICOM datasets were reviewed in multiplanar mode on an ENLIL PACS^®^ workstation (v2.5.32.b622, ENLIL Yazılım ve Bilişim A.Ş., Istanbul, Turkey). The “Adjust Window” tool was used to set the window level (WL) to 162 HU and the window width (WW) to 16 HU. This narrow window compresses the gray-scale mapping so that cortical bone densities above 170 HU appear as high-contrast white (255 gray level), while densities below 150 HU appear as black (0 gray level), thereby sharpening the bone margins. Windowing was performed solely via a lookup table (LUT) transformation; neither the underlying CT attenuation values nor the geometric resolution were modified.

Scanner calibration and QC: Daily air calibration and weekly water-phantom checks are performed on both scanners. A Catphan 500 phantom is run monthly; water HU remains within 0 ± 4, high-contrast MTF is ≥50% at 0.5 Lp/mm, and geometric distortion is <1 mm across a 200 mm field.

Native resolution and resampling: Helical data are reconstructed to 0.6 mm isotropic voxels (512 × 512 matrix, FOV 320–380 mm; in-plane pixel ≈ 0.63–0.74 mm). For surface roughness analysis, volumes are resampled to 0.5 mm^3^ using third-order B-spline interpolation to standardize point-cloud density.

Noise suppression and artefact handling: A 3 × 3 median filter suppresses quantum mottle. Beam-hardening streaks are mitigated with Siemens iMAR (level 2) and residual outliers removed by HU sigma-clipping (μ ± 3 σ). Scans showing ring artefacts wider than one pixel were discarded (*n* = 6).

Surface-extraction pipeline: (i) Threshold > 170 HU → (ii) marching-cubes mesh → (iii) Laplacian smoothing, 3 iterations, λ = 0.5 → (iv) PCA alignment to the vertebral mid-sagittal plane → (v) ellipse fitting to the superior endplate. All steps run in Python 3.10 (scikit-image 0.23); average processing time ≈ 9 s per case.

**Figure 1:**
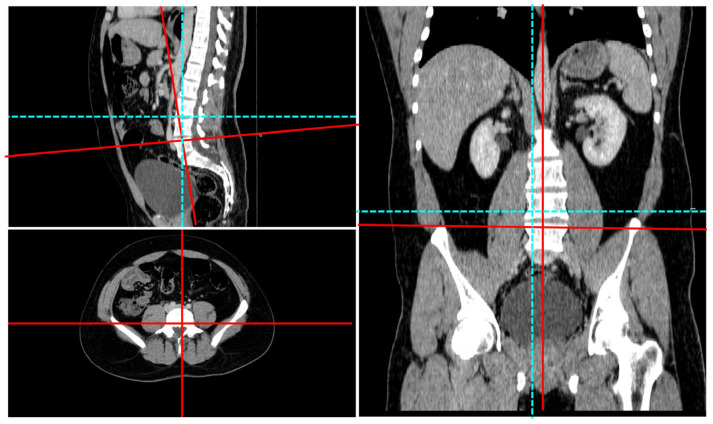
Multiplanar display of the CT image and adjustment of the planes according to the orientation of the superior endplate of the L5 vertebral body. The blue lines indicate the standard planes, while the red lines represent the adjusted planes.

For each of the 19 vertebral bodies from C7 through S1, the field of view was adjusted to the individual corpus orientation; an axial plane parallel to the superior endplate of each vertebra was identified, and that slice was exported for further analysis (Figure 1). A reference measurement was also recorded when saving each vertebral body’s superior endplate image to allow later standardization of measurements to millimeters.

### 2.4. Image Preparation

**Figure 2:**
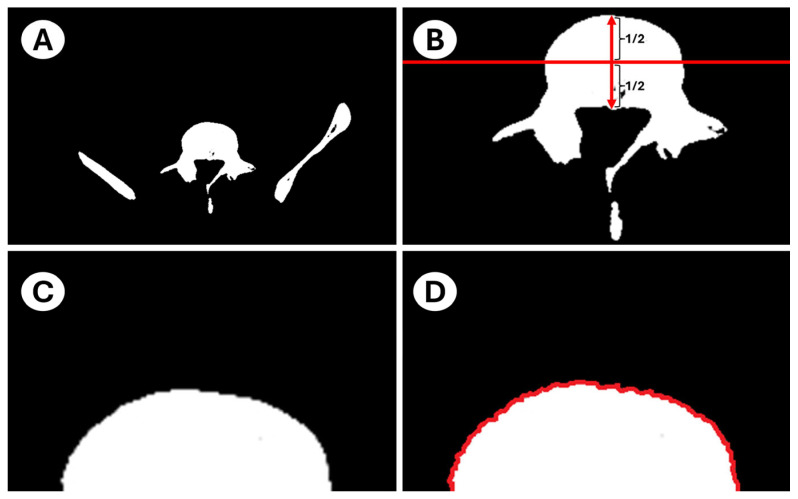
Image preparation workflow for isolating the anterosuperior vertebral endplate and preparing it for automated edge detection. (**A**) L5 vertebral body and surrounding osseous structures windowed at WL = 162 HU and WW = 16 HU to display only the bone contours. (**B**) Perpendicular reference line dividing the vertebral body into equal anterior and posterior halves; pedicles remain attached. (**C**) Isolated anterior half of the upper endplate, separated from all other vertebral elements. (**D**) Final anterosuperior endplate image with surface-edge coordinates extracted via automated edge detection.

Each image was then imported into the ImageJ^®^ (Version 1.54g, National Institutes of Health, Bethesda, MD, USA) environment [36]. Because the anterior half of the vertebral body more closely resembles an ellipse—and because the pedicles would interfere with subsequent automatic edge detection—only the anterior surface was analyzed. To that end, a perpendicular scale line was drawn to span the anteroposterior length of the vertebral body, and from its midpoint the anterior half of the image was isolated. The physical length of the reference line (in millimeters) was noted, then its pixel length was measured using ImageJ’s straight-line length tool; these values completed the image-scaling step. Finally, all other bony and vascular structures falling within the field of view were removed via freehand selection and cutting, yielding an isolated anterosuperior endplate image ready for automated edge detection and further processing (Figure 2).

### 2.5. Image Processing

Numerous image-processing techniques have been developed to quantify the roughness of a surface or profile [37,38,39]. The “ellipse-fit + mean deviation” approach was first reported for laser-PBF-fabricated micro-struts. Hossain et al. [39] fitted an ellipse to micro-CT-derived strut cross-sections using a least-squares algorithm and defined $S_{a}\;$as the mean absolute orthogonal distance of each network node from the ellipse; they demonstrated that this roughness score increased systematically with build angle.

In the present study, we adapted this concept to quantify the anterior vertebral end-plate roughness, drawing on the observation that the end-plate geometry in young adults approximates an ellipse. By using the idealized ellipse as a “young/healthy” reference surface, the mean deviation reduces complex surface irregularities to a single, reproducible quantitative indicator. This adaptation of Hossain et al.’s [39] method to bone biomechanics provides a novel metric for vertebral aging studies.

Image segmentation and automatic contour extraction were implemented entirely in Python 3 using the OpenCV and NumPy libraries as follows:

First, each CT slice was loaded as a matrix I(x,y) of 8-bit grayscale pixel intensities (0–255). Otsu’s method was then applied to determine an optimal threshold θ, producing a binary mask

(1) $$m\left(x,y\right)=\left\{\begin{array}{c} 1,\;\;I\left(x,y\right)\geq \theta ,\\ 0,\;\;I\left(x,y\right)<\theta . \end{array}\right.$$

Next, external contour points

(2) $$c={\left\{\left(x_{i},\;\;y_{i}\right)\right\}}_{i=1}^{L}$$

were extracted via cv2.findContours(…, cv2.RETR_EXTERNAL, cv2.CHAIN_APPROX_NONE) and only the largest contour—identified by ${arg\;max}_{c}\;Area\left(C\right)$ was retained to remove small noisy contours. To eliminate the effect of a perfectly horizontal baseline, we computed the most frequent contour y-coordinate

(3) y∗=argmaxyxi,yi ϵ C:yi=y

and filtered out all points lying within τ = 2 pixels of that line:

(4) $$C^{\prime }=\left\{\left(x_{i},y_{i}\right)\epsilon \;C:\;\left|y_{i}-y*\right|>\tau \right\}.$$

This step preserves only the true surface undulations for subsequent analysis. The extracted coordinate points for each vertebral image of every subject were saved in separate CSV files. These CSV datasets were then parsed and passed on to the subsequent ellipse-fitting procedure.

Ellipse fitting was implemented in a Python 3 environment using NumPy, pandas, matplotlib, and SciPy.optimize as follows:

Each coordinate CSV was read into a data matrix

(5) $$P\epsilon R^{n\times 2}$$

via pandas. A coarse initial estimate of the ellipse parameters—center $\left(x_{0},y_{0}\right)$, semi-axis lengths $a_{0}$ and $b_{0}$, and rotation angle $\varphi_{0}$—was obtained by performing PCA on the covariance of P.

To prepare for optimization, the semi-axis lengths were log-transformed while preserving the center and rotation information, yielding an initial parameter vector $q_{0}$.

**Figure 3:**
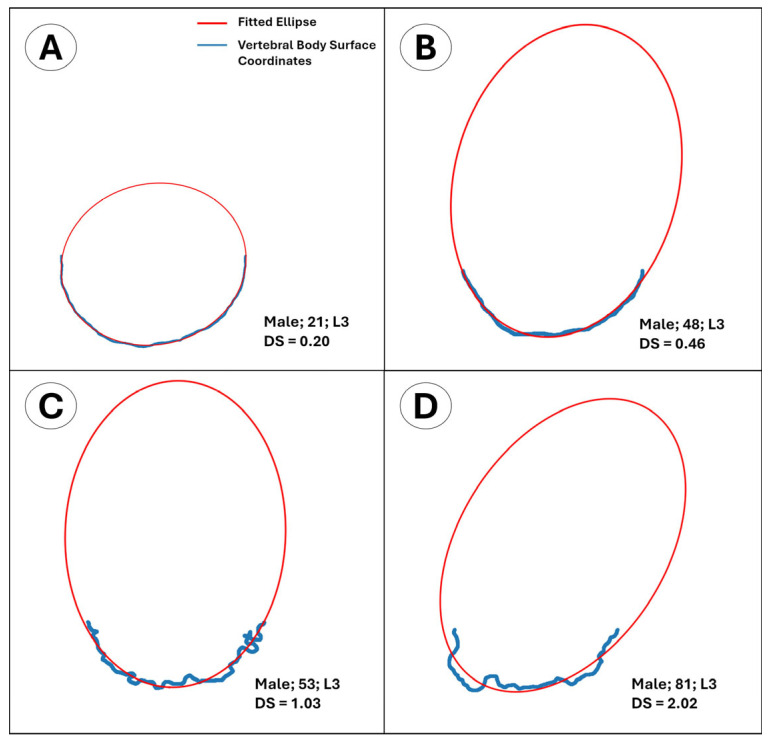
Surface-edge coordinate profiles of the isolated anterior half of the superior L3 vertebral endplate from four different male individuals, aged 21, 48, 53, and 81, respectively, overlaid with ellipses fitted by ellipse fitting algorithm. Note that as surface roughness increases (**A**–**D**), the average distance to the fitted ellipse (DS) also increases.

**Figure 4:**
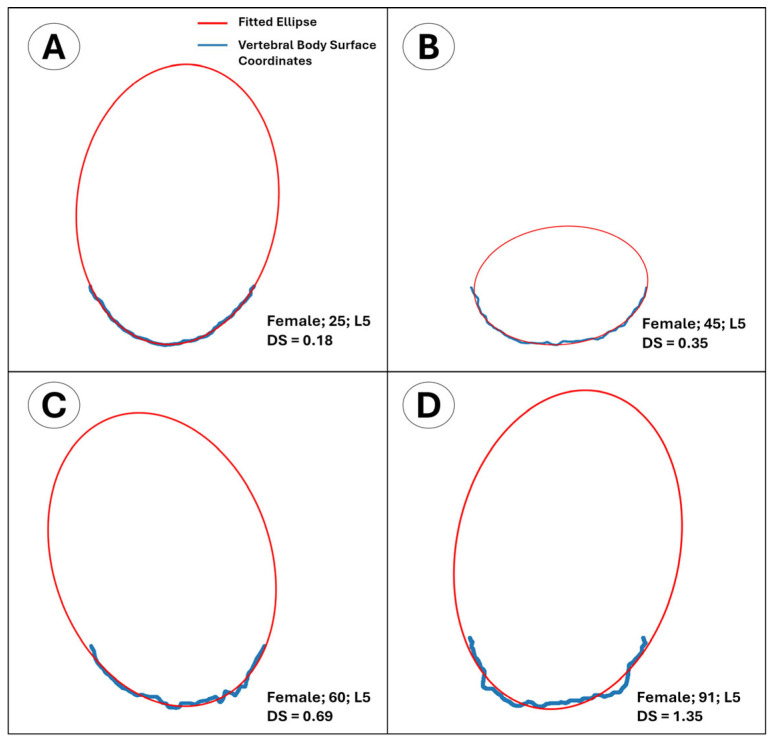
Adapted version of Figure 3 showing the surface-edge coordinate profiles of the isolated anterior half of the superior L5 vertebral endplate from four different female subjects aged 25, 45, 60, and 91. Similarly, as age—and therefore surface roughness—increases (**A**–**D**), the average distance to the fitted ellipse (DS) tends to increase.

The trust-region-reflective least-squares algorithm (least_squares (…, method = ‘trf’)) was used to minimize a composite residual function comprising: the ellipse equation residual

(6) $$r_{i}={\left(\frac{\left(x_{i}-x_{c}\right)cos\varphi +\left(y_{i}-y_{c}\right)sin\varphi }{a}\right)}^{2}+{\left(\frac{-\left(x_{i}-x_{c}\right)sin\varphi +\left(y_{i}-y_{c}\right)cos\varphi }{b}\right)}^{2}-1;$$

tangential penalties, which quantify how closely the contour endpoints satisfy the ellipse’s tangency condition; curvature penalties, enforcing adherence to the expected curvature at reference points; and an eccentricity constraint, penalizing any value of

(7) $$\log\left(\frac{b}{a}\right)$$

that exceeds a predefined threshold $r_{max}$. From the optimized parameters ($x_{c},y_{c},\;a,\;b,\;\varphi$), the nearest-point Euclidean distance of each contour point to the fitted ellipse was computed, and the mean error was reported as the average of these distances. The resulting mean error was adopted as a quantitative roughness index for the anterior surface of the superior endplate and termed the “average distance to the fitted ellipse” score (DS) (Figure 3 and Figure 4).

### 2.6. Statistical Analysis

The analyses were conducted in two complementary environments. First, IBM SPSS Statistics v27.0 (IBM Corp., Armonk, NY, USA) was used to screen the data for normality, identify extreme outliers (±3 SD), and compute Spearman correlation coefficients between chronological age and each of the 19 anterior-vertebral metrics.

All predictive models were then implemented in Python v3.10 (Python Software Foundation, Wilmington, DE, USA) with scikit-learn 1.4 and statsmodels 0.14. For each sex, the data were split once into an 80% training set and a 20% hold-out test set, after which 10-fold cross-validation (shuffled, random_state = 42) was applied inside the training portion for hyper-parameter tuning. Continuous predictors were z-standardized within every fold to avoid information leakage; tree-based algorithms, although scale-invariant, were embedded in the same pipeline for consistency.

Before modelling, the entire sample (*n* = 176) was subjected to an analysis of covariance (ANCOVA) in which chronological age was regressed on (1) sex as a fixed factor, (2) the 19 vertebral metrics as covariates, and (3) all first-order sex × metric interaction terms. The joint Wald test indicated that at least one sex-related coefficient differed significantly from zero (min *p* = 0.007). Consequently, all subsequent regressions were fitted separately for men and women. Within each sex, the modelling sequence began with multiple linear regression in SPSS to establish a classical benchmark (adjusted R^2^ and the standard error of the estimate, SEE). A LASSO model was then trained in Python; its α regularization parameter was selected from a log-spaced grid (10^−3^–10^1^) that minimized cross-validated mean absolute error. LASSO’s coefficient shrinkage provided a sparse predictor subset that guided the interpretation of non-parametric learners.

Four machine learning algorithms were evaluated in parallel. Support vector regression (SVR) mapped the scaled input into a high-dimensional Gaussian kernel space, where ε-insensitive loss produced a smooth regression surface; the cost (C), kernel width (γ), and ε parameters were grid-searched. Random forest (RF) regression aggregated 100–250 boot-strapped decision trees, with maximum depth varied to balance bias and variance; the method captures nonlinear interactions without explicit parametric assumptions. The k-nearest neighbors (KNN) regression provided a purely instance-based model in which each prediction is the mean age of the k nearest (Euclidean) neighbors; k was tuned between three and seven. Finally, a custom Gaussian naïve-Bayes regressor discretized age into equal-frequency bins, fitted a Gaussian-NB classifier to the binned labels, and projected its probabilistic output back to the continuous scale via bin mid-points—an approach suited to small samples when normality is plausible.

Model performance was quantified on the unseen test sets with mean absolute error (MAE), root mean squared error (RMSE), and the coefficient of determination (R^2^). In addition, the standard error of the estimate was reported, computed for every model as

(8) $$SEE=\sqrt{\frac{\sum_{i=1}^{n}\left(y_{i}-{ý}_{i}\right)}{n-k-1}}$$

where *n* is the number of cases in the relevant sex-specific subsample and k is the number of retained predictors (for LASSO, the non-zero coefficients). Separate SEE values facilitated direct comparison with earlier vertebral age-estimation studies that present sex-specific error bands. All analyses were executed with fixed random seeds and fully version-controlled scripts, ensuring complete reproducibility.

**Figure 5:**
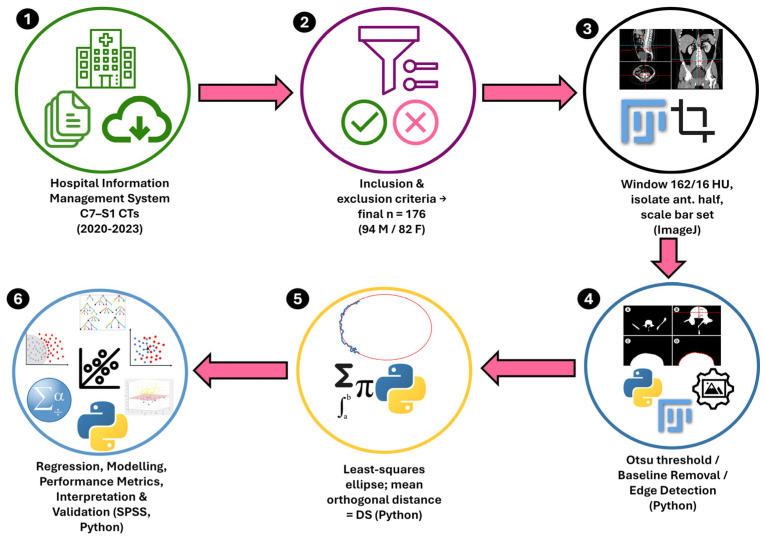
Six-step workflow of the DS-based vertebral age-estimation study. (**1**) Routine cervical-to-sacral (C7–S1) CT examinations performed between 2020 and 2023 were retrieved from the hospital information management system. (**2**) Inclusion and exclusion filters were applied, yielding 176 eligible adults (94 males, 82 females). (**3**) In ImageJ, the axial slice through each superior end-plate was windowed at 162/16 HU, the anterior half was isolated, and a scale bar was recorded. (**4**) A Python pipeline performed Otsu thresholding, baseline removal, and external contour extraction to obtain a clean cortical edge. (**5**) A least-squares ellipse was fitted to every contour; the mean orthogonal distance between contour nodes and the ellipse was calculated as the surface roughness score DS. (**6**) Sex-stratified linear, LASSO, and machine learning regressions (RF, KNN, SVR, GNB) were trained and evaluated; performance metrics and interpretation were generated in SPSS and Python.

A schematic overview of the entire six-step pipeline, from CT retrieval to statistical validation, is presented in Figure 5.

## 3. Results

### 3.1. Descriptive Statistics

The overall mean age of the sample was 58.5 years (median = 60.0; range = 21–95; SD = 13.9). Mean age was 56.9 years for men (median = 58.5; range = 21–85; SD = 13.2) and 60.2 years for women (median = 60.0; range = 25–95; SD = 14.7). The age distributions of male and female participants did not differ significantly (*p* = 0.128) (Appendix A Figure A1, Figure A2 and Figure A3).

Table 1 presents sex-specific descriptive statistics for the DS. With the exception of the upper thoracic levels, the central tendency and spread of DS values are broadly similar in men and women (e.g., C7: 0.33 ± 0.34 vs. 0.35 ± 0.34).

Statistically significant sex differences are confined to a contiguous mid-thoracic band—T3 through T6, as well as T11, T12, and L1—where women show systematically lower roughness than men (mean gap ≈ 0.05–0.08 DS units). No significant differences emerge in the lumbar bodies L2–L5 or in S1, and variability (min–max range and SD) is comparable across sexes except at T5 and L3, whose female distributions are widened by a few high-roughness outliers. These findings indicate that sexually dimorphic surface ageing is most pronounced in the mid-thoracic region, whereas lumbar roughness—key to the predictive models—does not differ between males and females.

### 3.2. Correlation Between Age and Vertebral Surface Roughness (Table 2; Figure 6 and Figure 7)

In males, 11 of the 19 DS variables correlated significantly with age (*p* < 0.05). The strongest association was observed at L3 (r = 0.60, *p* < 0.001), followed by L4, L2, S1, and L5 (r = 0.40–0.45, *p* < 0.001). Mid-thoracic roughness (T8–T11) showed weaker but still meaningful correlations (r ≈ 0.32–0.35), whereas DS for the upper thoracic and cervical levels (C7, T1–T5) was essentially unrelated to age (r ≤ 0.13, *p* > 0.20).

Female results displayed the same cranio-caudal gradient but with uniformly higher coefficients: 15 DS variables were significant, the top five again belonging to the lumbar spine—L5 (r = 0.59), L2, L4, L3, and L1 (all r ≥ 0.49, *p* < 0.001). Thoracic DS at T8–T11 ranked next (r ≈ 0.36–0.52). Cervical (C7) and upper-thoracic (T1) scores remained nonsignificant, mirroring the male findings. Of note, the sacral base (S1) correlated with age in males (r = 0.40, *p* < 0.001) but not in females (*p* = 0.51).

Taken together, the results demonstrate a moderate-to-strong, sex-specific monotonic increase in vertebral surface roughness—quantified by DS—from the mid-thoracic region down to the lumbar spine, with the lumbar levels providing the clearest chronological signal.

### 3.3. Multiple Linear Regression (Table 3)

The sex-specific linear models explained a moderate share of age variance. In women, the 19 DS predictors jointly accounted for 47% of the variability (adjusted R^2^ = 0.304) with a standard error of the estimate (SEE) of 12.3 years, whereas the male equation explained 40% (adjusted R^2^ = 0.244; SEE = 11.4 years). Both regressions were highly significant (F = 2.8–2.6, *p* ≤ 0.002).

Collinearity diagnostics indicated that redundancy among the vertebral DS scores was mild and well below critical thresholds in both sexes: the highest variance-inflation factor was 3.22 (tolerance = 0.311) in females and 2.36 (tolerance = 0.424) in males, and the largest condition indices were 17–18 (far short of 30). Consequently, no predictor was removed.

At the individual coefficient level, none of the female DS terms reached the 0.05 threshold, suggesting that age information is distributed across several lumbar and lower-thoracic sites rather than concentrated in a single level. In the male model, only the L3 score (β = 0.43, *p* = 0.002) remained significant after mutual adjustment, reinforcing the univariable finding that the caudal lumbar surface contributes most strongly to male age estimation. Despite these differences in term-level significance, the overall SEE values for both sexes lie within the 11–12-year band that is considered acceptable in previous vertebral age-estimation studies.

### 3.4. LASSO Regression

LASSO regression—with the same optimal penalty (α = 2.06) for both sexes—reduced the 19-level DS panel to a very compact set of age predictors. In men, the algorithm retained only the upper-lumbar surfaces, L3 (β = 4.27) and L2 (β = 0.68); this two-variable equation explained 30% of the test-set variance (R^2^ = 0.30) with a root mean square error of 11.2 years (SEE = 12.2 y on the test fold; 11.4 y when refitted to the full sample). In contrast, the female solution was more diffuse, keeping eight coefficients—four lower-thoracic (T2, T4, T8, T9) and four lumbar (L1, L2, L4, L5)—all positively signed, with T8 and T9 carrying the largest weights (β ≈ 2.0). This broader lumbar-plus-thoracic set captured 47% of the test variance (R^2^ = 0.47), yielding a test SEE of 13.5 years (12.5 y on the combined dataset). Thus, while LASSO confirmed the primacy of lumbar roughness in males, it highlighted a mixed thoracolumbar ageing pattern in females; in both cases, the shrinkage approach produced SEE values comparable to—and slightly lower than—the corresponding multilinear models, but with far fewer parameters.

### 3.5. Machine Learning Models (Table 4)

The discretized Gaussian naïve-Bayes (GNB) baseline performed worst in the male series, with a hold-out R^2^ = −1.15 (95% CI −4.30 to −0.13) and a standard error of the estimate (SEE) of 17.0 y (MAE 14.5 y, RMSE 17.0 y). Internal 5 × 5 cross-validation (CV MAE 10.3 y; CV R^2^ = −0.12) had predicted markedly smaller errors, confirming that the algorithm memorized the development set and systematically over-estimated younger males and under-estimated older ones.

The k-nearest neighbor (KNN) regressor provided an intermediate male result: hold-out R^2^ = 0.26 (95% CI −0.79 to 0.66) with SEE = 10.0 y (MAE 7.1 y, RMSE 10.0 y). This is ≈ 2 years more precise than GNB. A slightly higher CV R^2^ = 0.37 indicates a mild but acceptable optimism gap; KNN narrows the error margin by ~1 year relative to multiple linear regression.

After nested tuning (C = 100, γ = 0.01, ε = 0.1), the radial-kernel SVR (RBF-SVR) achieved a hold-out R^2^ = −0.18 (95% CI −1.37 to 0.32) and SEE = 12.7 y (MAE 10.4 y, RMSE 12.6 y). Although CV R^2^ rose to 0.44, the independent test split revealed substantial over-fitting and only marginal improvement over linear regression.

The linear-kernel SVR yielded modest explanatory power: hold-out R^2^ = 0.12 (95% CI −0.70 to 0.41) with SEE = 10.9 y (MAE 8.8 y, RMSE 10.9 y). A similar CV R^2^ = 0.10 shows the model captures little nonlinear structure and performs between KNN and RBF-SVR.

The random forest (RF) ensemble delivered the best male accuracy, achieving a hold-out R^2^ = 0.47 (95% CI −0.01 to 0.67) and SEE = 8.5 y (MAE 6.7 y, RMSE 8.5 y). Internal validation was consistent (CV R^2^ = 0.43; OOB R^2^ = 0.45), indicating stable generalization with minimal optimism. Compared with KNN, the forest reduces the standard error by ~1.5 years and almost doubles explained variance, confirming it as the preferred male learner.

In the female sample, discretized GNB again failed to capture the ageing signal: hold-out R^2^ = −0.20 (95% CI −1.77 to 0.33) with SEE = 15.9 y (MAE 14.0 y, RMSE 15.9 y). A slightly negative CV R^2^ = −0.08 confirms that poor generalization stems from model bias rather than data scarcity.

The female KNN explained 45% of the external variance (hold-out R^2^ = 0.45; 95% CI −0.26 to 0.77) with SEE = 10.8 y (MAE 7.98 y, RMSE 10.8 y) and a CV R^2^ = 0.304. It halves the SEE relative to GNB and represents the current benchmark for women.

The female RBF-SVR offered only marginal benefit: hold-out R^2^ = 0.10 (95% CI −0.69 to 0.42) with SEE = 13.8 y (MAE 10.9 y, RMSE 13.8 y). CV R^2^ = 0.31 suggests a small optimism gap, yet the model remains ~3 years less precise than KNN and only slightly better than GNB.

The female linear SVR was weaker still, returning a negative hold-out R^2^ = −0.27 (95% CI −2.10 to 0.41) and SEE = 16.4 y (MAE 12.1 y, RMSE 16.4 y). With CV R^2^ = 0.15, it ranks at the bottom of the female hierarchy.

The female random forest provided a middle-ground solution: hold-out R^2^ = 0.22 (95% CI −0.74 to 0.73) and SEE = 12.8 y (MAE 9.1 y, RMSE 12.8 y). Internal metrics were stronger (CV R^2^ = 0.374; OOB R^2^ = 0.418), indicating honest generalization. The forest trims the SEE by ~3 years compared with RBF-SVR and ~4 years compared with GNB or linear SVR yet remains ≈ 2 years less precise than KNN.

In sum, random forest is the clear winner for men, whereas KNN remains the top performer for women. SVR variants offer only incremental gains, and both naïve-Bayes and linear SVR are unsuitable as stand-alone forensic tools.

Across the male sample, diagnostic panels converged on a clear performance hierarchy. Random forest (RF) emerged as the only algorithm that balanced bias and variance: L3–L4 dominated its permutation importance profile, residuals were tight and unbiased, and learning curves showed high but not perfect training R^2^ (~0.9) with steadily rising CV R^2^, yielding the narrowest limits of agreement (≈±17 y). K-nearest neighbor (KNN) relied on a broader lumbar-plus-lower-thoracic pattern; its residuals were symmetric and homoscedastic, but a plateauing CV R^2^ (~0.25) revealed variance limitation, placing KNN as a serviceable—but clearly inferior—fallback to RF. In contrast, both support vector regressors were hampered by bias: the RBF kernel latched onto upper-lumbar levels, dramatically over-aged young men and under-aged older ones, and displayed a steep residual slope, while the linear kernel compressed predictions into a 50–65 y band, leaving large age-dependent errors; their learning curves showed either near-perfect memorization (RBF) or persistent under-fit (linear). The discretized Gaussian naïve-Bayes (GNB) fared worst: it effectively used a single mid-lumbar predictor and produced age “plateaus,” a funnel-shaped residual pattern, bimodal error distribution, and extreme over-fitting (train R^2^ > 0.95, CV R^2^ < 0.10). Taken together, RF is the only model with real forensic promise, KNN provides a bias-free but variance-limited alternative, both SVR variants add little value, and GNB serves only as a comparative baseline to be discarded in practice.

In the female cohort, diagnostics again revealed a clear pecking order: k-nearest neighbor (KNN) remained the standout, explaining ~45% of external variance with the narrowest error band (SEE ≈ 10.8 y) and minimal age-dependent bias, its learning curve indicating variance—not bias—limits that further data could remedy. The random forest (RF) ensemble offered the next-best trade-off: cross-validated R^2^ approached 0.40, residuals were near-normal and largely unbiased (median ≈ −1 y), and although one extreme outlier inflated the upper Bland–Altman limit, overall dispersion (SEE ≈ 12.8 y) was ~3 y tighter than either support vector model; permutation importance highlighted a diffuse upper-thoracic/upper-lumbar pattern (T7, T9, L4) rather than the male L3/L4 focus. Both support vector regressors were hampered by bias: the RBF kernel compressed ages into 55–75 y, under-ageing the oldest woman and widening limits to ±27 y, while the linear kernel fared worst—predictions squeezed into 50–75 y, negative hold-out R^2^ (−0.27), and the broadest limits (≈−38 y to +27 y). Finally, the Gaussian naïve-Bayes (GNB) baseline catastrophically over-fitted (train R^2^ ≈ 0.9, CV ≤ 0), yielded bimodal residuals and limits of ±26 y, underscoring its unsuitability. In sum, KNN remains the only truly forensic-grade option for women at present, RF provides a robust but slightly less precise alternative, both SVR variants offer marginal utility, and GNB is best retained solely as a comparative benchmark. The learning curve panels, Bland–Altman plots, permutation importance plots, predicted vs. true scatter plots, residual vs. fitted plots, and residual histograms for both male and female cases are presented in detail—and interpreted—in Appendix A.

### 3.6. Post Hoc Power Analysis

Using the observed coefficients of determination from the multiple linear regression benchmarks (Table 3) and the exact sample sizes of the final data sets (male *n* = 94, female *n* = 82), we calculated the achieved power for the overall regression F-test. With 19 predictors, the denominator degrees of freedom were 74 (males) and 61 (females). Converting the observed fits to effect-size indices

(9) $$\left(Cohen’s\;f^{2}=\frac{R^{2}}{\left[1-R^{2}\right]}\right)$$

yielded f^2^ = 0.66 for men and 0.88 for women. The corresponding non-centrality parameters

(λ = f^2^ · df_2_) (10)

were 49.1 and 53.9. At α = 0.05, the critical F value is ≈ 1.73; the non-central F distribution therefore gives an achieved power of 0.992 for the male model and 0.995 for the female model. In other words, the study had >99% probability of detecting the observed age-related variance if it was present in the source population.

However, the power to estimate individual regression coefficients or to generalize the SEE to new samples is appreciably lower. Each sex provides only ~4.3 (male) and ~3.9 (female) observations per predictor, well below the commonly recommended 10–15 cases per variable. This shortfall is reflected in the wide 95% confidence intervals of the SEE, the learning curve analyses, and the large permutation importance uncertainties (Appendix A).

## 4. Discussion

### 4.1. Synopsis of the Main Findings

Accurately estimating adult age or age at death remains one of the most persistent challenges in forensic anthropology, clinical forensic medicine, forensic radiology, and bio-archaeology because most protocols still depend on semi-subjective scoring of heterogeneous degenerative features. In the present study, we propose a novel alternative to subjective scoring: a single, objective descriptor of vertebral cortical roughness—the mean orthogonal deviation of each vertebral endplate from an ideal ellipse (DS)—calculated automatically for C7–S1 on routine multi-detector CT scans. DS increases progressively from the mid-thoracic to the lumbar spine and correlates moderately to strongly with chronological age, particularly at L3–L5, where r values reach 0.49–0.60 in women and 0.40–0.60 in men. When DS values from all 19 vertebrae were entered into multivariable models, classical multiple regression accounted for 40% of the age variance in men and 47% in women (SEE ≈ 11–12 years). Non-parametric algorithms improved precision: a random forest ensemble reduced the male SEE to 8.49 years, whereas a k-nearest neighbors model lowered the female SEE to 10.8 years (R^2^ = 0.448 on the hold-out set). Thus, the automated DS metric provides age estimates comparable to those generated by more complex pipelines. These findings indicate that a simple, reproducible measure of cortical roughness obtainable from routine clinical images can serve as an alternative indicator of skeletal aging.

### 4.2. How DS Compares with Conventional Vertebral Scores

The standard error of the estimate (SEE) achieved by the present DS models compares favorably with most vertebral-based aging studies published to date. Using a 20% hold-out set, the best female model (k-nearest neighbors) reached an SEE of 10.8 years, whereas the best male model (random forest) yielded 8.49 years; traditional multiple linear regression of the same predictors performed less well (≈12 years in females and 11 years in males). These figures place our automated method near the forefront of CT-derived vertebral methods while requiring neither deep-learning architectures nor specialized hardware.

When set against classical osteophyte or surface score approaches, the DS metric offers a clear precision advantage. Regional osteophyte-based models derived from Japanese autopsy CTs [21] and Thai dry vertebrae [19] typically report SEE values of ~10–11 years, while inspection-style indices such as Watanabe’s [17] whole-column score and Zangpo’s [26] 3-D surface-deformation metric cluster between 11 and 13 years (Table 5). Using the same linear regression framework, our DS predictor produces SEE values in a comparable range (≈11–12 years). However, when DS is coupled with non-parametric algorithms—k-nearest neighbors in females and random forests in males—the SEE falls to 10.8 years and 8.49 years, respectively (Table 4). Although these errors are roughly one-third to one-half lower than the classical figures, the improvement partly reflects the greater flexibility of machine learning learners rather than an inherent superiority of DS alone. Indeed, more advanced models in earlier studies (e.g., Schanandore’s [31] semi-quantitative lumbar RMSE ≈ 8.4 years) narrow the gap, emphasizing that numerical comparisons should be interpreted with caution whenever statistical platforms differ. Nevertheless, the present results demonstrate that DS contains sufficient signal to equal or exceed the precision of most traditional vertebral indicators under like-for-like modelling, and that its accuracy can be substantially enhanced by applying readily available non-parametric methods.

### 4.3. DS Versus Deep Learning Pipelines

Deep learning pipelines currently set the numerical gold standard. Kawashita et al.’s [24] whole-spine bagged VGG−16 ensemble achieved an SEE of 5.5 years on a cadaver validation set, and Nurzynska et al.’s [23] texture-based CNN reported an MAE of 3.1 years. While our female SEE lies within roughly one year of the Kawashita figure, the male error remains higher. Nonetheless, the DS approach attains this level of accuracy with an interpretable single-feature paradigm, minute preprocessing, and conventional CPUs—traits that may facilitate deployment in resource-constrained forensic or clinical settings.

### 4.4. Population-, Sex-, and Age-Specific Patterns

Sample composition remains a critical—and often under-reported—determinant of external validity in vertebral aging research. The most accurate pipelines to date have been trained and validated almost exclusively within a single ethnic group: the bagged VGG-16 ensemble of Kawashita et al. [24] used only Japanese clinical and post-mortem CTs, while the texture analysis study of Nurzynska et al. [23] relied on Polish cardiac patients. By contrast, several osteophyte-based investigations draw on Thai dry bone [19] collections that are markedly male-skewed (≈65% male), whereas Adams et al.’s [22] radiographic series was deliberately balanced at 50% per sex. Our cohort of 176 living Eastern Turkish adults (94 males, 82 females) therefore fills a geographic gap, introducing Middle Eastern representation while maintaining a near-even sex split. The broader biogeographic coverage is valuable for future meta-analyses and may help test whether the DS roughness metric is truly ethnicity-agnostic or, like many macromorphological scores, demands population-specific calibration. Replicating the DS workflow in independent African, East Asian, and European samples—ideally with comparable sex balance—will clarify whether universal decision thresholds can be set or whether regional standardization is still required to achieve forensic-grade accuracy.

**Figure 6:**
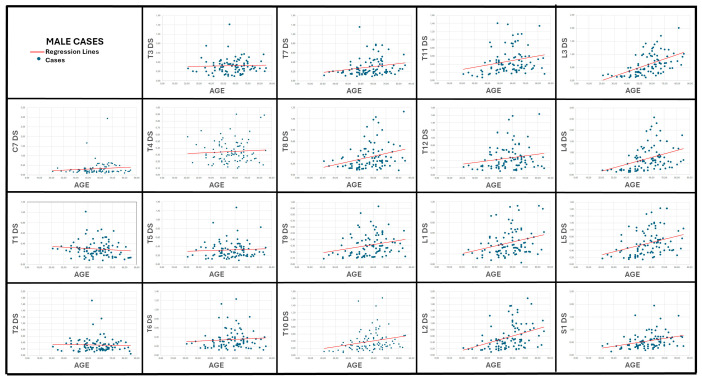
Scatter plots and regression lines depicting the relationship between age and the average distance-to-fitted-ellipse (DS) values computed for each of the 19 vertebrae from C7 to S1 in male cases.

**Figure 7:**
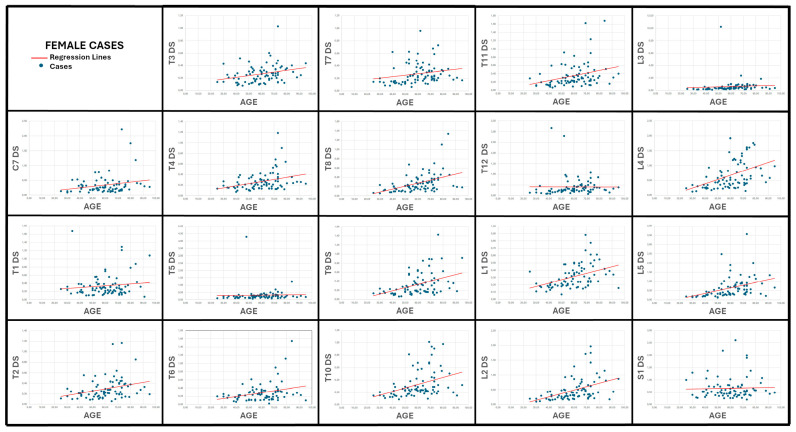
Scatter plots and regression lines depicting the relationship between age and the average distance-to-fitted-ellipse (DS) values computed for each of the 19 vertebrae from C7 to S1 in female cases.

Osteophyte-based formulas typically lose traction in very old adults because osteophyte height approaches a biological ceiling after about 70 years [17,25]. By contrast, the regression scatterplots generated for our study (Figure 6 and Figure 7) reveal that lumbar DS values continue to rise across the full adult age span, with a clearly positive slope that persists even among the small cluster of octogenarians. Consistent with that visual trend, the overall Pearson correlations reported in Table 2 remain moderate for L3–L5 (≈0.40–0.60 in men and 0.49–0.60 in women), indicating that cortical roughness keeps accruing after osteophyte growth has largely stabilized. Consequently, the DS-based models do not show the marked loss of precision in seniors that is characteristic of height-limited osteophyte equations, supporting the view that surface roughness trajectories provide a more informative signal of late-life skeletal ageing.

Lumbar vertebrae consistently carry the strongest chronological signal in vertebral-aging research: for example, the Thai dry bone study of Praneatpolgrang et al. [19] reported its highest age correlations in the lumbar mean score (r = 0.76), and our own permutation importance map for males likewise places L3–L5 at the top of the ranking. In women, however, the DS metric shows a broader “thoraco-lumbar” footprint—moderate correlations extend from T8 through L5 (Table 2). This sex-specific topography may reflect biomechanical and hormonal factors: pregnancy-related shifts in lumbar lordosis, lower-thoracic rib cage expansion, and post-menopausal estrogen decline all redistribute loading and remodeling rates along the spine, potentially accelerating cortical scalloping in the mid-thorax for females while concentrating it in the lower lumbar bodies for males. A practical consequence of this anatomical breadth is workflow burden. The image preparation step of our proposed method is labor intensive, and the time required limited our sample size. Future studies could streamline the protocol by targeting only the vertebrae that show the strongest age associations in each sex (e.g., T8–L5 in women, L3–L5 in men) or by using deep learning segmentation to automate the image preparation step.

### 4.5. Model Diagnostics and Practical Performance

Classic vertebral age protocols rest on observer-assigned scores—for example, the five-stage Snodgrass [15] or the four-stage Watanabe [17] osteophyte scales—whose inter-observer agreement rarely exceeds an intraclass correlation coefficient of ≈ 0.85 even after calibration sessions [25]. Such subjectivity poses a reproducibility risk when multiple laboratories must defend estimates in court. By contrast, the present study uses an automated contour-fit algorithm to extract the DS roughness metric, eliminating rater bias and allowing the same result to be regenerated from the raw DICOMs at any site.

From a modelling standpoint, our linear regression baseline (SEE ≈ 11–12 years) mirrors the precision of most classical regression formulas, isolating the intrinsic signal contained in DS. When we switched to shallow, non-parametric learners—random forests for men and k-nearest neighbors for women—the SEE dropped to 8.49 years and 10.8 years, respectively, demonstrating the added value of algorithmic flexibility without sacrificing interpretability. Deep learning systems such as the bagged VGG-16 ensemble of Kawashita et al. [24] push the SEE down to ≈ 5.5 years, but they do so at the cost of “black-box” decision rules and high GPU demand. The DS + shallow-ML approach occupies a transparent middle ground: it matches or modestly surpasses the SEE reported for manual osteophyte-based formulas (≈11–13 y; see Table 5) while clearly outperforming our own classical linear baseline (≈11–12 y), yet remains computationally light, fully explainable, and readily portable. Because manual scores were not applied to the present CT set, this comparison is indirect and warrants verification in a future head-to-head study.

### 4.6. Practical Significance of the Results

Because the method presented here is implemented with open-source code, can be readily applied to routinely acquired vertebral CT scans, and—with future development—could be deployed as a fully automated, user-friendly software, it holds practical utility across several key areas. First, in forensic anthropology practice—especially in jurisdictions that routinely employ postmortem computed tomography (PMCT) on unidentified remains [40]—analysts could automatically survey the entire vertebral column to generate an initial age estimate, thereby guiding and potentially reducing the need for subsequent, more advanced, costly, or destructive sampling procedures. Even an age range estimated with an error margin of ±8–10 years can markedly narrow the pool of potential matches. Furthermore, this method can be extended to other bones and teeth, and, by employing an ensemble approach, the error margin can be further reduced. Second, in clinical forensic contexts—such as elite sport eligibility determinations or asylum cases [41,42]—this method could be applied to living individuals for age estimation. In particular, the availability of existing trauma CT scans in medical archives would allow application of this technique without additional radiation exposure. Finally, this method could be fully automated and extended to additional skeletal elements to enhance predictive accuracy, then integrated into hospital information management systems, thereby enabling routine use by clinicians. Because it requires neither high computational power nor GPUs, this method can be readily implemented in hospital systems without imposing an undue burden.

### 4.7. Population and Sex Variation and Taphonomic Considerations

While the present study focused exclusively on a contemporary Turkish clinical population, the issue of population variability remains a central concern in age estimation research. Previous work has shown that skeletal aging trajectories can differ substantially across geographic and ethnic groups due to genetic, environmental, and cultural influences [15,30]. Although our dataset helps address the underrepresentation of Middle Eastern populations, external validation in diverse cohorts remains necessary to assess whether the DS metric generalizes across populations or requires region-specific calibration. With respect to sex-related differences, our results show that while lumbar roughness patterns dominate age prediction in males, females exhibit a more diffuse thoraco-lumbar pattern. This topographic difference may reflect biomechanical factors (e.g., pregnancy-induced lordosis, rib cage remodeling) or hormonal changes, especially postmenopausal bone turnover [15,43,44]. These differences are captured both in correlation profiles (Table 2) and in the variable selection paths of the sex-specific models (Section 3.4 and Section 3.5). Finally, because our sample consisted exclusively of living adults undergoing diagnostic CT scans, postmortem taphonomic alterations—such as microbial degradation, desiccation, or soil pressure—that might affect surface roughness [35,45,46] were not present in these images. While this design avoids the noise introduced by such variables, it also limits generalizability to forensic remains. Future work should replicate this pipeline on postmortem CT datasets, which may introduce new sources of surface distortion or artefacts that affect the DS score.

### 4.8. Ethical and Legal Implications of Bone Surface Roughness-Based Age Estimation

Bone surface roughness—as determined by DS calculations from CT images in this study or acquired from dry skeletal remains using structured light scanning (SLS) [47]—qualifies as biometric data under the EU General Data Protection Regulation [48,49]. Therefore, when such data is to be used in forensic medical research, particularly in cases involving minors, asylum seekers, or deceased individuals, it may be necessary to obtain consent from appropriate legal guardians or next of kin before proceeding with examinations. Another ethical and legal concern relates to the probabilistic nature of adult age estimation. The DS-based pipeline, like all current adult age estimation techniques, yields a probabilistic estimate rather than a deterministic determination [50]. Even at our best standard error of the estimate (SEE ≈ 8 years), cases will inevitably fall outside the ±8-year confidence band, and misclassification may have substantial legal or humanitarian consequences (e.g., asylum eligibility, criminal liability thresholds). When age opinions are presented in court, experts therefore have an ethical obligation to (i) report the method’s quantified error margins, (ii) state that the result is an estimate rather than a factual age, and (iii) caution decision makers against using the point estimate in isolation [51].

### 4.9. Pathophysiological Basis of the DS Metric

The progressive increase we observe in the DS score has a clear histo-morphological foundation. With advancing age, the anterior-superior vertebral cortex is subjected to rising compressive and shear loads—especially after disc height loss [52]—causing repetitive micro-fracture [53], osteoclastic resorption, and subsequent remodeling [54]. Histological and µCT studies demonstrate a linear rise in cortical porosity [55], Haversian canal enlargement [56], and surface scalloping throughout adulthood [54], while trabecular bone volume fraction and connectivity decline in parallel [57]. These processes generate two macroscopic signatures that the DS algorithm captures: (i) outward protrusions (osteophytic ridges, ligamentous ossification) that shift boundary nodes beyond the “young/healthy” ellipse; and (ii) inward indentations (resorption pits, cortical thinning) that pull nodes inside the ellipse. The DS measure is therefore an integrated geometric read-out of both productive (osteophyte formation) and destructive (porosity-driven undermining) phases of vertebral aging. Importantly, these alterations accumulate even after osteophyte height reaches its biological ceiling [17,25], explaining why lumbar DS continues to climb in octogenarians whereas height-based scores plateau. Hence, the metric is not a black-box surrogate but a quantifiable expression of well-documented degenerative pathways, reinforcing its biological plausibility and forensic relevance.

## 5. Limitations

The principal limitation is the sample size relative to the model complexity. Although post hoc power for the global regression is high, the ratio of observations to predictors raises the risk of over-fitting, inflates coefficient sampling error, and limits the precision of the SEE—especially in cross-validation and external test sets (SEE 95% CI up to ±3 years). Second, the cohorts originate from a single clinical center and share similar ethnic and socio-economic backgrounds; extrapolation to other populations therefore warrants caution. These constraints should be addressed in future studies by enlarging the sample and validating the method on independent populations. Because classical osteophyte/degeneration scores were not re-assessed on the same CT sample, our comparison with “traditional” methods remains indirect; a future head-to-head study is therefore required to confirm the incremental accuracy of the DS + shallow-ML approach.

## 6. Conclusions

This study introduces an automated “average distance to the fitted ellipse” score (DS) descriptor of vertebral cortical roughness and demonstrates its utility for adult age estimation on routine multi-detector CT scans spanning C7–S1. DS correlates positively with chronological age across the thoraco-lumbar spine and, when entered into sex-specific machine learning models, yields SEE values of 10.8 years in females (k-nearest neighbors) and 8.49 years in males (random forest)—demonstrating comparable accuracy relative to classical multiple linear regressions (≈11–12 years) and most published osteophyte-based formulas. Because the pipeline is observer independent, light on hardware, and fully reproducible from raw DICOM data, it offers a transparent alternative to subjective scoring systems and data-hungry deep learning solutions. Nevertheless, several limitations warrant attention: the preparation of 19 vertebral images per subject is time consuming; the sample derives from a single Turkish center; and model generalizability beyond the studied ethnicity remains untested. Future work should automate the image preparation step, explore whether a reduced vertebral subset can retain accuracy, and validate the DS approach in larger, multi-ethnic cohorts.

## Figures and Tables

**Table 1:** Sex-specific descriptive statistics for distance to fitted ellipse roughness score.

Variable	Male (*N* = 94)				Female (*N* = 82)				*p*
	Mean ± SD	Median	Min–Max	95% CI	Mean ± SD	Median	Min–Max	95% CI	
C7 DS	0.33 ± 0.34	0.24	0.09–2.93	0.26–0.40	0.35 ± 0.34	0.26	0.09–2.23	0.28–0.43	0.917
T1 DS	0.30 ± 0.15	0.27	0.08–1.02	0.27–0.33	0.34 ± 0.28	0.27	0.08–1.68	0.28–0.40	0.991
T2 DS	0.33 ± 0.22	0.26	0.06–1.73	0.28–0.37	0.30 ± 0.20	0.24	0.06–1.17	0.25–0.34	0.136
T3 DS	0.33 ± 0.16	0.30	0.10–1.22	0.29–0.36	0.27 ± 0.15	0.25	0.08–1.03	0.24–0.30	0.005 *
T4 DS	0.35 ± 0.16	0.31	0.10–0.90	0.32–0.38	0.27 ± 0.18	0.22	0.08–1.19	0.23–0.31	0.000 *
T5 DS	0.33 ± 0.18	0.28	0.09–1.28	0.29–0.36	0.32 ± 0.47	0.24	0.09–4.30	0.22–0.42	0.022 *
T6 DS	0.35 ± 0.20	0.31	0.12–1.24	0.30–0.39	0.29 ± 0.23	0.24	0.02–1.54	0.24–0.34	0.003 *
T7 DS	0.30 ± 0.19	0.23	0.10–1.16	0.26–0.34	0.27 ± 0.17	0.23	0.06–0.96	0.24–0.31	0.374
T8 DS	0.32 ± 0.22	0.25	0.08–1.13	0.27–0.36	0.28 ± 0.21	0.22	0.06–1.34	0.23–0.32	0.135
T9 DS	0.31 ± 0.17	0.27	0.09–0.94	0.27–0.34	0.33 ± 0.25	0.23	0.07–1.43	0.28–0.39	0.801
T10 DS	0.39 ± 0.29	0.30	0.08–1.62	0.33–0.45	0.33 ± 0.22	0.25	0.09–1.01	0.28–0.38	0.073
T11 DS	0.48 ± 0.30	0.40	0.08–1.41	0.41–0.54	0.36 ± 0.30	0.27	0.07–1.68	0.29–0.42	0.000 *
T12 DS	0.46 ± 0.31	0.39	0.11–1.64	0.40–0.52	0.41 ± 0.46	0.28	0.08–3.18	0.31–0.51	0.021 *
L1 DS	0.41 ± 0.25	0.33	0.03–1.13	0.36–0.46	0.31 ± 0.16	0.25	0.07–0.88	0.28–0.35	0.009 *
L2 DS	0.57 ± 0.39	0.46	0.10–1.79	0.49–0.65	0.49 ± 0.38	0.35	0.10–1.97	0.41–0.58	0.112
L3 DS	0.62 ± 0.40	0.50	0.13–2.02	0.54–0.70	0.62 ± 1.13	0.41	0.12–10.25	0.38–0.87	0.094
L4 DS	0.74 ± 0.55	0.61	0.13–2.58	0.63–0.85	0.68 ± 0.47	0.53	0.12–1.93	0.58–0.78	0.633
L5 DS	0.61 ± 0.36	0.52	0.15–1.63	0.54–0.68	0.65 ± 0.56	0.47	0.13–3.57	0.53–0.78	0.622
S1 DS	0.56 ± 0.31	0.47	0.15–1.96	0.50–0.63	0.66 ± 0.43	0.53	0.20–2.61	0.57–0.76	0.079

DS: the score of average distance to fitted ellipse. *: *p* value < 0.05; there is a statistically significant difference between the two sex groups in terms of the relevant variable.

**Table 2:** Correlation between chronological age and vertebral metrics in males and females.

	Males		Females	
Variable	r	*p*	*r*	*p*
C7 DS	0.089	0.395	0.194	0.081
T1 DS	−0.108	0.301	0.096	0.392
T2 DS	−0.039	0.711	0.355	<0.01 (*)
T3 DS	0.078	0.453	0.334	<0.01 (*)
T4 DS	0.014	0.893	0.362	<0.001 (**)
T5 DS	0.059	0.575	0.314	<0.01 (*)
T6 DS	0.129	0.214	0.287	<0.01 (*)
T7 DS	0.279	<0.01 (*)	0.352	<0.01 (*)
T8 DS	0.324	<0.01 (*)	0.522	<0.001 (**)
T9 DS	0.322	<0.01 (*)	0.459	<0.001 (**)
T10 DS	0.352	<0.001 (**)	0.471	<0.001 (**)
T11 DS	0.284	<0.01 (*)	0.367	<0.001 (**)
T12 DS	0.175	0.092	0.324	<0.01 (*)
L1 DS	0.280	<0.01 (*)	0.495	<0.001 (**)
L2 DS	0.424	<0.001 (**)	0.553	<0.001 (**)
L3 DS	0.600	<0.001 (**)	0.509	<0.001 (**)
L4 DS	0.448	<0.001 (**)	0.538	<0.001 (**)
L5 DS	0.395	<0.001 (**)	0.588	<0.001 (**)
S1 DS	0.403	<0.001 (**)	0.074	0.508

* *p* < 0.01; ** *p* < 0.001; DS: the score of average distance to the fitted ellipse.

**Table 3:** Multiple linear regression of average distance to the fitted ellipse scores (DS) on chronological age.

Statistic	Females (*n* = 81)	Males (*n* = 94)
R	0.685	0.631
R^2^	0.469	0.399
Adjusted R^2^	0.304	0.244
SEE (years)	12.29	11.44
F (df)	2.84 (19, 61)	2.58 (19, 74)
Model *p*	0.001	0.002
Max VIF (variable)	3.22 (L2 DS)	2.36 (T8 DS)
Min tolerance (variable)	0.311 (L2 DS)	0.424 (T8 DS)
Max condition index	17.3	17.6
Collinearity flag	none	none

**Table 4:** Performance of four machine learning regressors for age prediction.

Sex	Model	Holdout R^2^	95% CI (R^2^)	SEE (y)	95% CI (SEE)	MAE (y)	RMSE (y)	CV R^2^	OOB R^2^ †
Male	RF	0.468	−0.01–0.67	8.49	5.8–10.9	6.69	8.49	0.432	0.446
Male	SVR-rbf	−0.184	−1.37–0.32	12.65	9.0–15.8	10.39	12.65	0.442	—
Male	SVR-lin	0.118	−0.70–0.41	10.92	7.6–14.0	8.77	10.92	0.098	—
Male	KNN	0.257	−0.79–0.66	10.03	6.3–13.5	7.12	10.03	0.367	—
Male	GNB-Reg	−1.147	−4.30–−0.13	17.04	13.0–21.0	14.46	17.04	−0.120	—
Female	RF	0.221	−0.74–0.73	12.83	5.9–18.4	9.06	12.83	0.374	0.418
Female	SVR-rbf	0.097	−0.69–0.42	13.81	9.6–17.5	10.85	13.81	0.311	—
Female	SVR-lin	−0.271	−2.10–0.41	16.39	9.9–22.5	12.11	16.39	0.153	—
Female	KNN	0.448	−0.26–0.77	10.80	6.3–14.8	7.98	10.80	0.304	—
Female	GNB-Reg	−0.202	−1.77–0.33	15.94	11.9–19.5	14.04	15.94	−0.080	—

R^2^ = coefficient of determination; SEE = standard error of the estimate; MAE = mean absolute error; RMSE = root mean squared error; CV R^2^ = mean 10-fold cross-validated R^2^ calculated on the 80% training split; OOB R^2^ = out-of-bag R^2^ (internal validation metric computed only for random forest); CI (95%) = two-sided percentile bootstrap confidence interval based on 1000 resamples of the external test set; RF = random forest regressor; SVR = support vector regression; KNN = k-nearest neighbors regressor; GNB-Reg = discretized Gaussian naïve-Bayes pseudo-regressor. † Out of Bag R^2^ is calculated only for the Random Forest model.

**Table 5:** Overview of vertebral-based adult age estimation methods: cohorts, imaging modalities, modeling approaches, and reported accuracy.

**Reference**	**Population and Sample Size**	**Vertebral Region and Imaging/Data**	**Age Indicators and Modelling Approach**	**Reported Performance †**	**Key Contribution/Remarks**
Adams et al., 2024 [22]	240 medico-legal cases (120 males and 120 females; 18–101 y)	Lower T-spine and upper L-spine; digital radiographs	3-phase degenerative score of T11–L3 → Bayesian transition analysis	Bin-1 → <36 y, Bin-3 → >47 y (90% CI); no sex effect	Practical radiographic protocol for fleshed remains; usable when skeletal sampling impossible.
Cardoso & Ríos 2011 [18]	104 documented skeletons (47 males and 57 females; 9–30 y)	Cervical, thoracal, and lumbar vertebrae; dry bone	3-stage scoring of epiphyseal-ring union	Stage 0 always < 18 y; stage 3 appears ≥ 15 y	Detailed fusion timetable for adolescent vertebrae; fills gap for 10–25 y age window.
Chiba et al., 2022 [21]	250 PMCT cadavers (125 females and 125 males; 20–95 y)	T- and L-spine; post-mortem CT	Osteophyte score O (0–5) + bridge score B (0–2); regression	Best SEE ≈ 10 y using “number of vertebrae with O ≥ 2”	Demonstrates CT-based scoring viable even with partial columns.
					
**Reference**	**Population and sample size**	**Vertebral region and imaging/data**	**Age indicators and modelling approach**	**Reported performance †**	**Key contribution/remarks**
Etli 2023 [34]	140 Turkish CT cases (70 males and 70 females; 21–90 y)	Sacral base; abdominal–pelvic CT	Distance-to-fitted-ellipse surface roughness score (DS); sex-specific linear regression	MAE 12.5–14.8 y, RMSE 14.7–18.1 y; DS ≥ 50 y class accuracy >80%	Pilots the DS concept, showing moderate precision with sacral DS.
Garoufi et al., 2022 [28]	275 Europeans (168 Greek modern; 93 males and 75 females; 107 Danish archaeological; 56 males and 51 females; 21–82 y)	T12 superior and inferior end-plates; digital photographs	9 geometric variables → generalized additive regression	Max. R^2^ 0.46; correct-decade hit-rate 33% (archaeol. set)	Introduces continuous 2-D shape metrics; moderate age signal, highlighted size-related sex effects.
Kaçar et al., 2017 [25]	564 living Turkish adults (279 males and 285 females; 20–84 y)	(T1–L5); 0.5 mm MDCT scans reconstructed to 3-D volume-rendered images	Vertebral osteophyte severity (0–4 scale); Linear regression for age estimation (upper/lower limits).	Significant age correlation (40–70 yrs, both sexes). Sex-specific thoracic/lumbar age formulas (*p* < 0.05). Inter/intra-rater reliability: 0.85/0.88.	First large CT study on living adults; shows osteophyte severity peaks in mid-thoracic region and plateaus after 70 years; detects male-biased osteophyte frequency at T9–T12; provides practical formulas for forensic age estimation.
Kawashita et al., 2024 [24]	Training: 1120 clinical CTs; 560 males and 560 females; 20–99 y; Test: 219 PMCT cadavers; 137 males and 82 females; 21–94 y	Whole spine; axial CT slices → VGG-16 regression ensemble	Deep learning regression (bagged VGG-16)	MAE = 4.36 y; SEE = 5.48 y; ICC = 0.96	First end-to-end DL model on spine; accuracy surpasses classic scores, robust to 20–90 years.
Malatong Y et al., 2022 [27]	Thai skeletal radiograph bank; 220 lumbar DR images (110 males, 110 females; 20–86 y)	L1–L5; posterior–anterior digital radiographs	Image-analysis of trabecular “black-pixel” content: total % (TP), mean % (MP), black/white ratio (BW); stepwise linear regression by sex	Best equations: male (L4) SEE 15.4 y, female (L1) SEE 13.8 y; r = 0.21–0.46	Introduces automated pixel-density metric as surrogate for bone porosity on plain films.
Nurzynska et al., 2024 [23]	166 routine axial CTs (95 males and 71 females; 20–80 y)	L-vertebral bodies; axial CT ROIs	(a) qMaZda texture features + ML regression; (b) custom CNN	Texture-ML: MAE 3.14 y, R^2^ 0.79; CNN slightly worse	Shows grey-level texture alone can predict age within ±3 y on moderate dataset.
Praneatpolgrang et al., 2019 [19]	400 Thai skeletons (262 males and 138 females, 22–97 y)	C2–L5; dry bone	Length-based osteophyte score (modified Snodgrass/Watanabe)	Lumbar female: r = 0.80; SEE ≈ 10–11 y (author-reported)	Provides full sex-/region-specific equations for tropical Southeast-Asian population.
Ramadan et al., 2017 [29]	123 clinical CTs (61 males and 62 females, 10–64 y)	Last thoracic (T12); MDCT axial slices	15 linear dimensions; stepwise multiple regression	Sex-classification 88.6%; age r ≤ 0.40, SEE not reported	Shows T12 size grows with age but correlation too weak for precise aging; useful primarily for sexing.
Rizos et al., 2024 [30]	219 documented Greeks (121 males and 98 females; 19–99 y)	64 skeletal traits incl. vertebral osteophytes; macroscopic scoring	Deep randomized neural network ensembles	MAE ≈ 6 y in >50 y group; poor (<10%) correct-decade in <50 y	Independent test questions “universal” accuracy. claims; stresses population-specific training.
					
**Reference**	**Population and sample size**	**Vertebral region and imaging/data**	**Age indicators and modelling approach**	**Reported performance †**	**Key contribution/remarks**
Schanandore et al., 2024 [31]	North-American medical CT archive; 319 scans (149 males and 170 females; 10–89 y)	T12–L5; clinical CT (0.6–1.3 mm slices); 3-D surface models in Mimics^®^	Six-point semi-quantitative osteophyte score on superior and inferior margins of each level (0–2); single and multiple linear regressions, 6-fold CV ×100	Best models (L1–L5 mean or multi-level totals): RMSE ≈ 8.4 y, R^2^ 0.85; 73–77% of cases ± 10 y; ICC 0.80–0.95	First to subdivide each margin; demonstrates lumbar (esp. L4) dominance; high accuracy with simple scores.
Sluis et al., 2022 [13]	88 19th-c. Dutch skeletons (40 males and 48 females; 19–90 y)	Full spine; dry bone	Mean osteophyte stage by three published methods	Correct age bin assignment 73–76%	External validation of three scoring schemes; supplies Dutch-specific regressions.
Snodgrass 2004 [15]	384 Terry Collection cases (192 males and 192 females; 20–80 y)	T- and L-spine; dry bone	5-stage osteophyte scale; sex comparison	Greater variability in female; recommends wider CI	Found broadly parallel aging curves; underscores need for sex-specific intervals.
Suwanlikhid et al., 2018 [33]	250 Thai dry vertebral columns (125 males and 125 females; 22–89 y)	L1–L5 macroporosity, cortical roughness, osteophytes; naked-eye scoring	Multiple linear regressions per surface	Best: L1-inferior surface R^2^ 0.41, SEE 11.7 y	Simple portable method; advocates combining three degenerative traits for tropical skeletal sets.
Thomsen et al., 2015 [16]	80 cadaver pairs (39 males and 41 females; 19–96 y)	L2 and iliac crest; μCT 3-D	Trabecular BV/TV, Tb.Th, SMI etc. vs. age	No explicit SEE; shows linear BV/TV decline (r ≈ −0.8)	Highlights microstructural trajectories; useful explanatory context for imaging biomarkers.
Watanabe & Terazawa 2006 [17]	225 Japanese autopsy cases (138 males and 87 females; 20–88 y)	Whole column; direct inspection and palpation	0–3 height-based scores averaged to “osteophyte index”; sex-specific regression	SEE 12.6 y (M)/11.9 y (F); r ≈ 0.70	Classic benchmark for simple inspection-based ageing in East Asian population.
Zangpo et al., 2023 [26]	200 Japanese PMCT cases (126 males/74 females, 25–99 y)	L4 body; 3-D PMCT surface mesh vs. convex-hull volumes	VR = mesh/hull; VD = (hull–mesh)/mesh; simple regressions	SEE 11.9 y (M)/12.5 y (F); ρ = ±0.76	First to quantify age from global 3-D surface bulging/concavity rather than marginal osteophytes.
Zangpo et al., 2024 [32]	200 Japanese PMCT cases (same cohort as 2023 study)	L4 body; max Hausdorff-distance (maxHD) between mesh and smoothed template	maxHD vs. age; sex-specific linear regression	SEE 12.5 y (M)/13.1 y (F); ρ ≈ 0.74	Confirms 3-D surface-deformation signal with an intuitive shape-difference metric (HD).

† SEE = Standard Error of the Estimate; MAE = Mean Absolute Error; RMSE = Root-Mean-Square Error; r = Pearson Correlation; ρ = Spearman Correlation.

**Figure A4:**
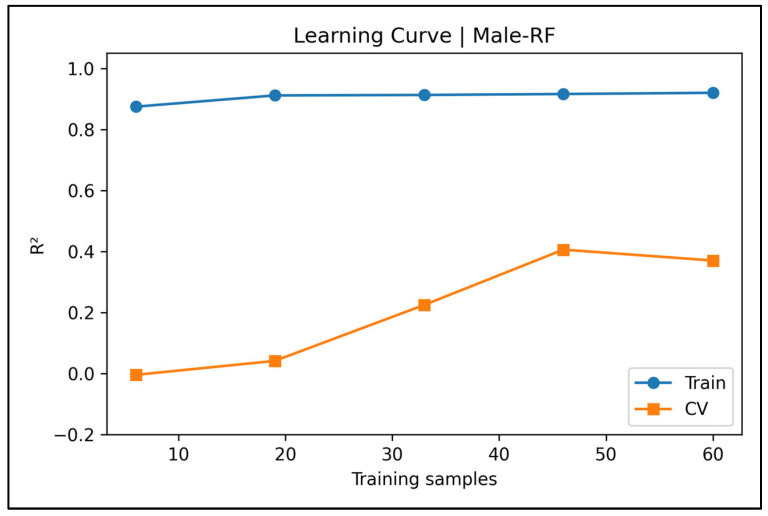
Learning curve for the male random forest model.

**Figure A5:**
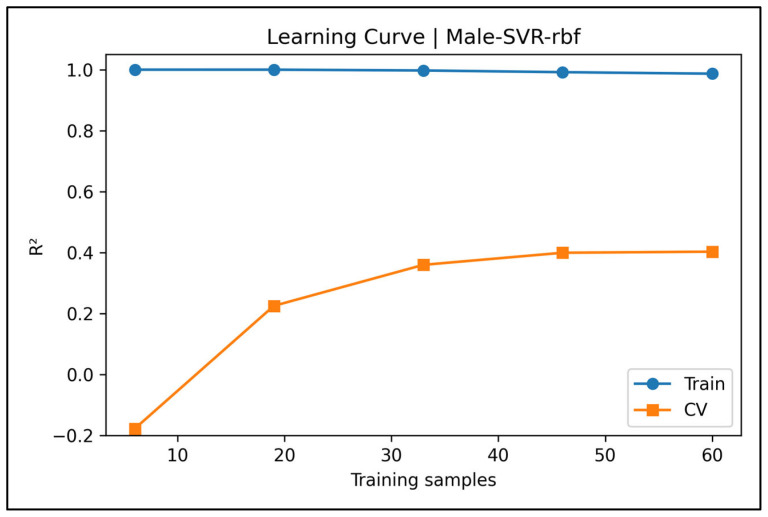
Learning curve for the male support vector regression (radial basis function) model.

**Figure A6:**
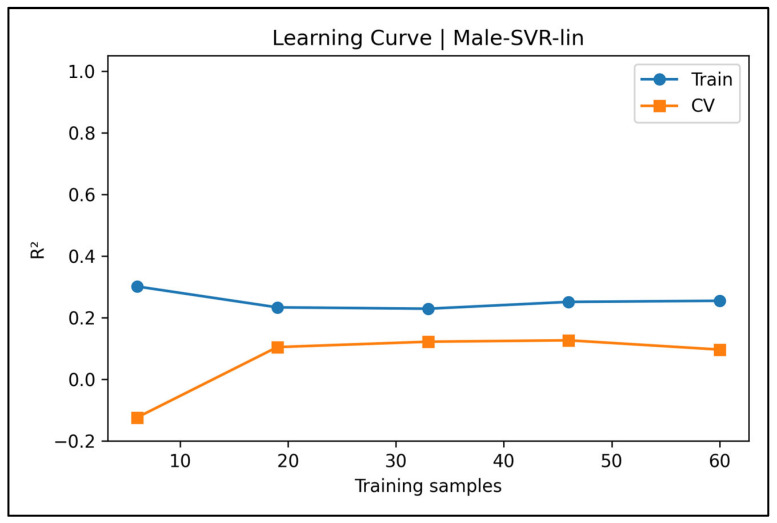
Learning curve for the male support vector regression (linear) model.

**Figure A7:**
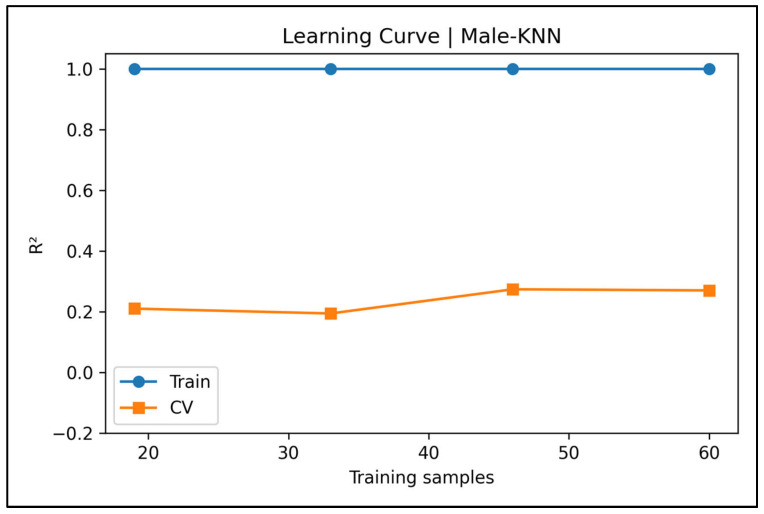
Learning curve for the male k-nearest neighbors model.

**Figure A8:**
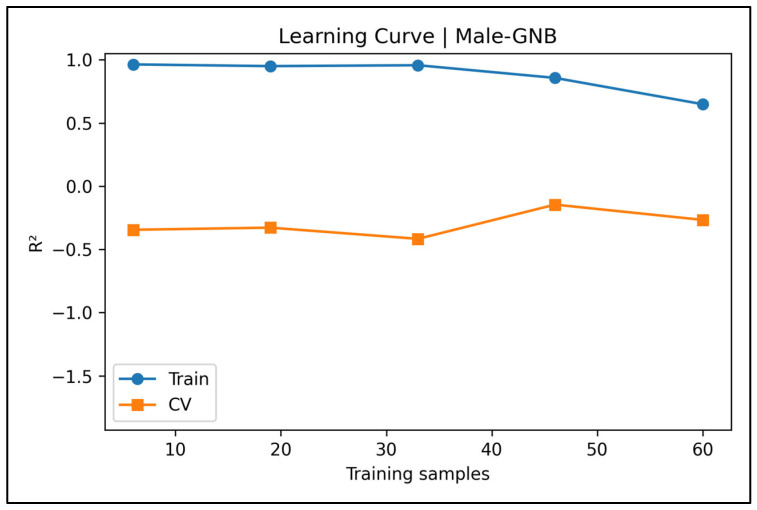
Learning curve for the male Gaussian naïve-Bayes regressor.

**Figure A9:**
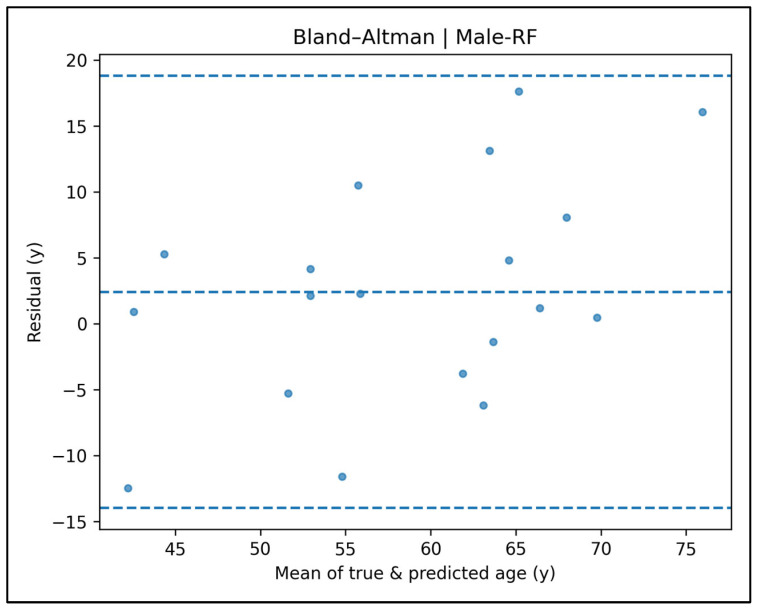
Bland–Altman plot for the male random forest model.

**Figure A10:**
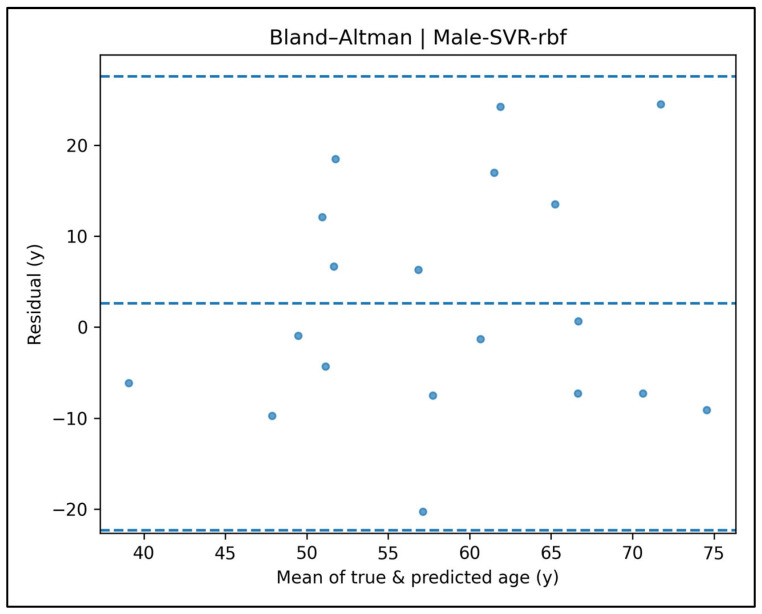
Bland–Altman plot for the male support vector regression (radial basis function) model.

**Figure A11:**
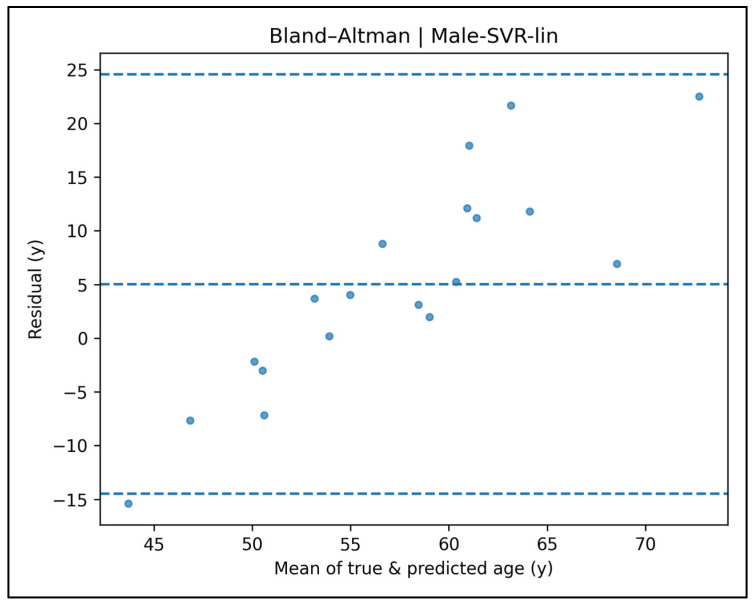
Bland–Altman plot for the male support vector regression (linear) model.

**Figure A12:**
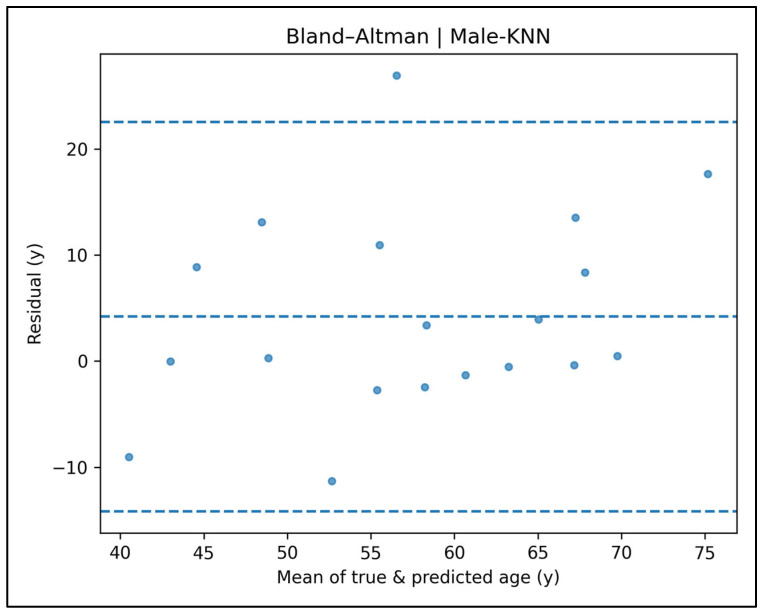
Bland–Altman plot for the male k-nearest neighbors model.

**Figure A13:**
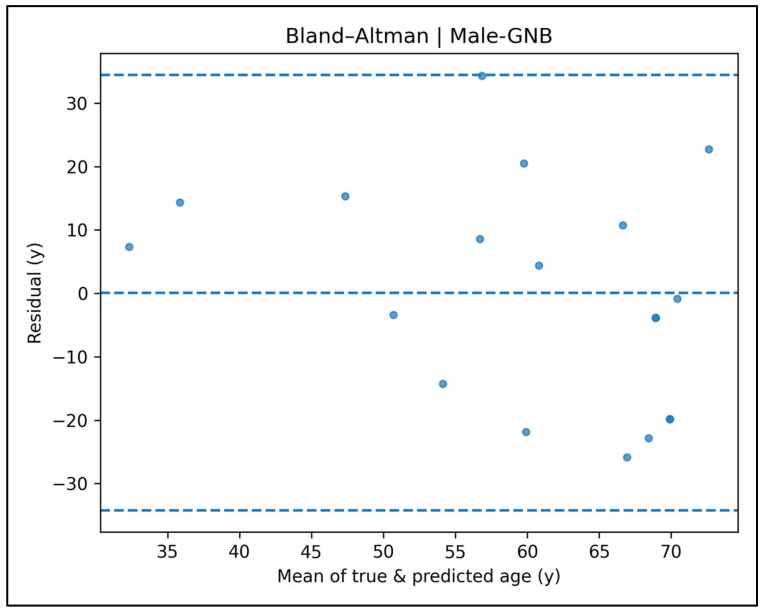
Bland–Altman plot for the male Gaussian naïve-Bayes regressor.

**Figure A14:**
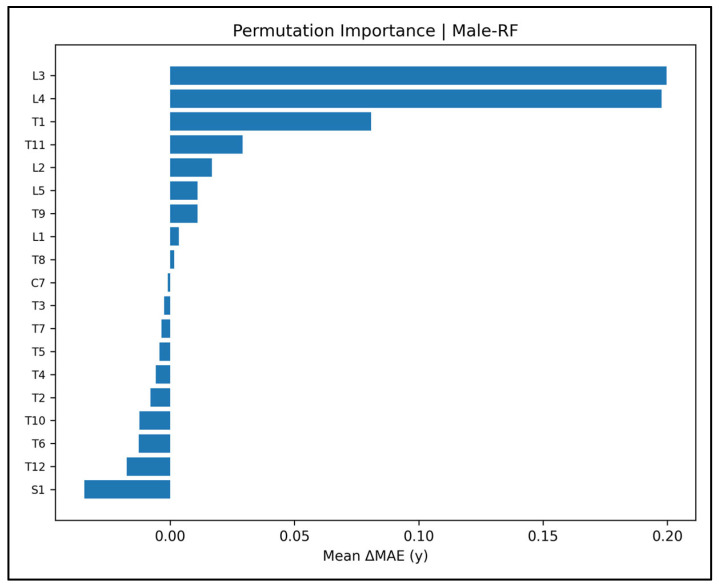
Permutation importance plot for the male random forest model.

**Figure A15:**
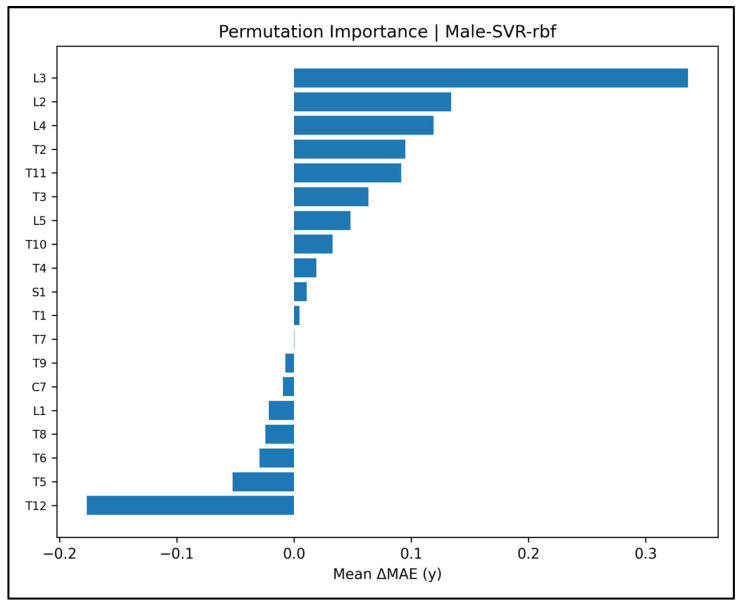
Permutation importance plot for the male support vector regression (radial basis function) model.

**Figure A16:**
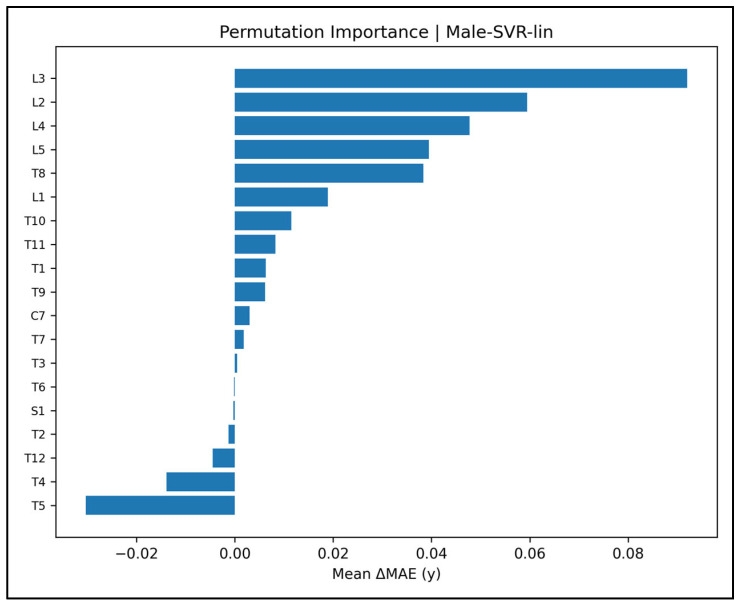
Permutation importance plot for the male support vector regression (linear) model.

**Figure A17:**
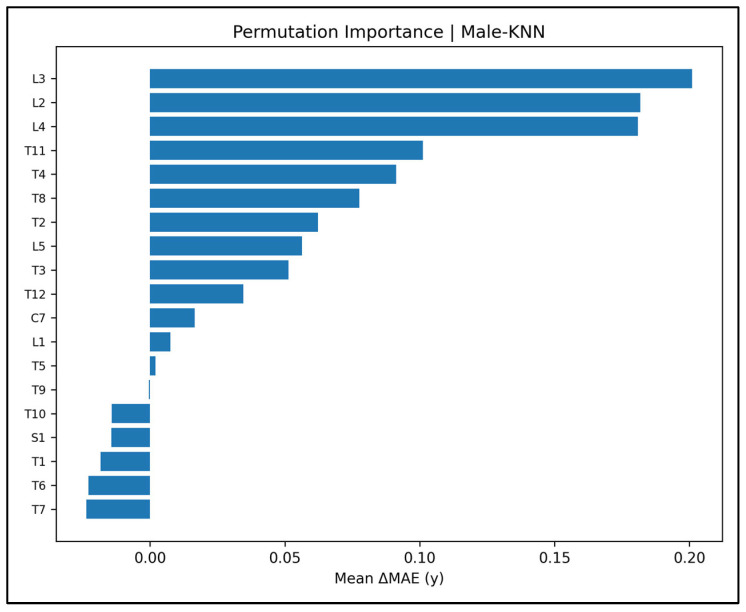
Permutation importance plot for the male k-nearest neighbors model.

**Figure A18:**
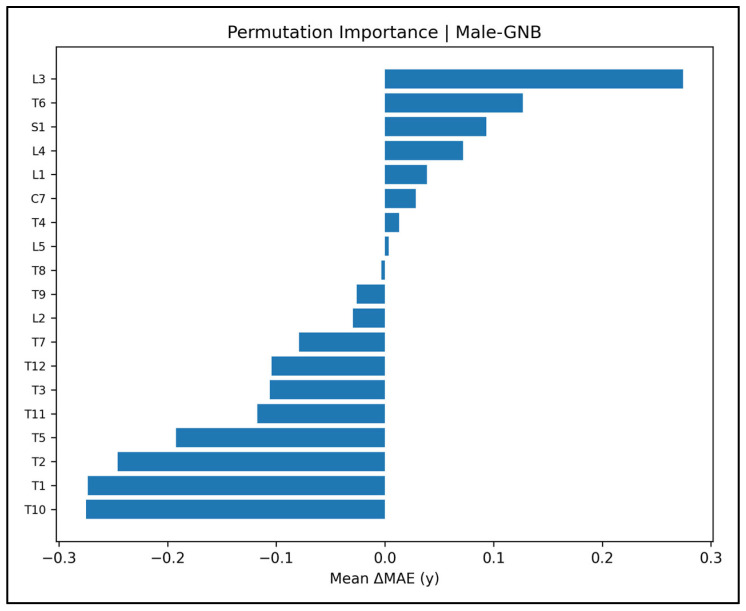
Permutation importance plot for the male Gaussian naïve-Bayes regressor.

**Figure A19:**
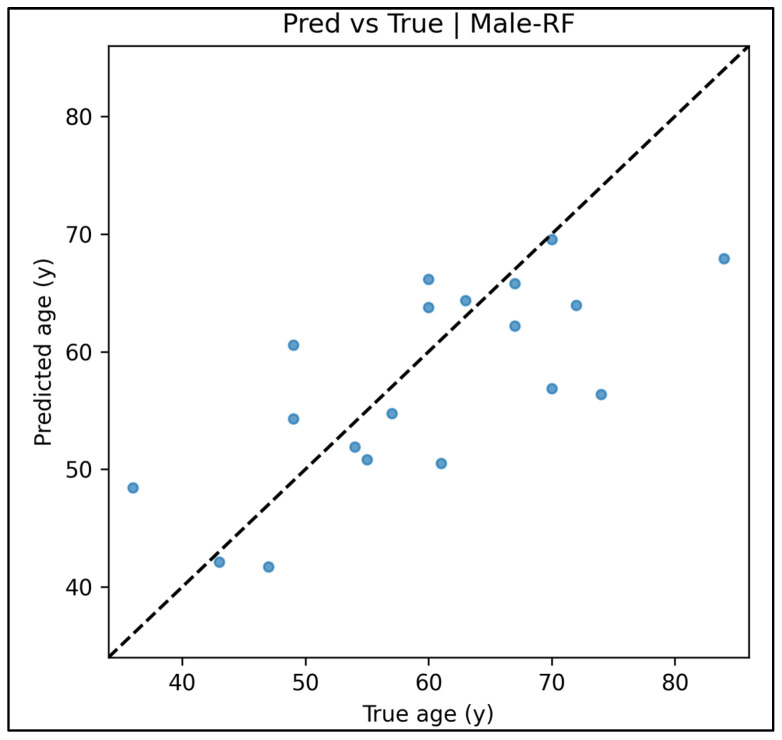
Predicted vs. true scatter plot for the male random forest model.

**Figure A20:**
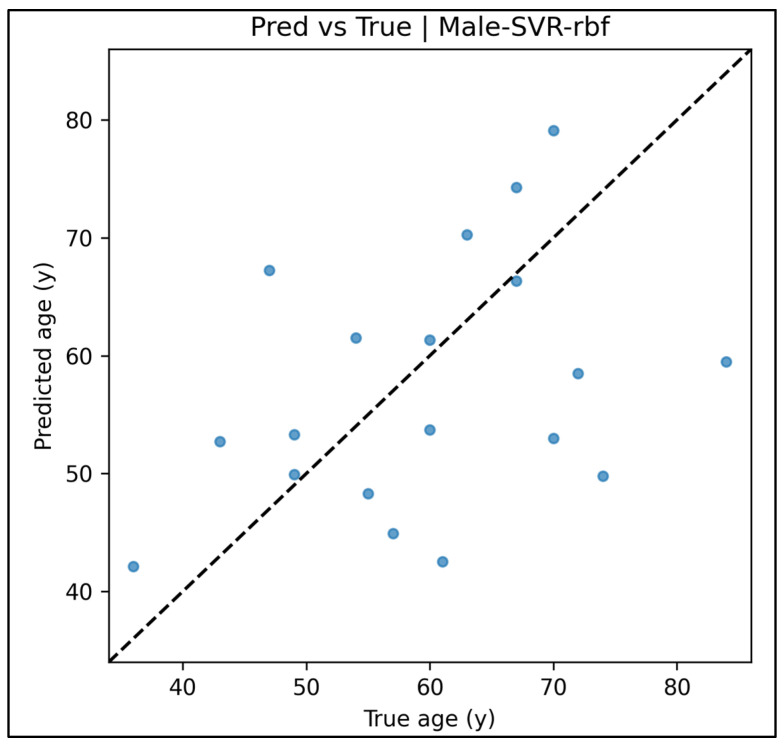
Predicted vs. true scatter plot for the male support vector regression (radial basis function) model.

**Figure A21:**
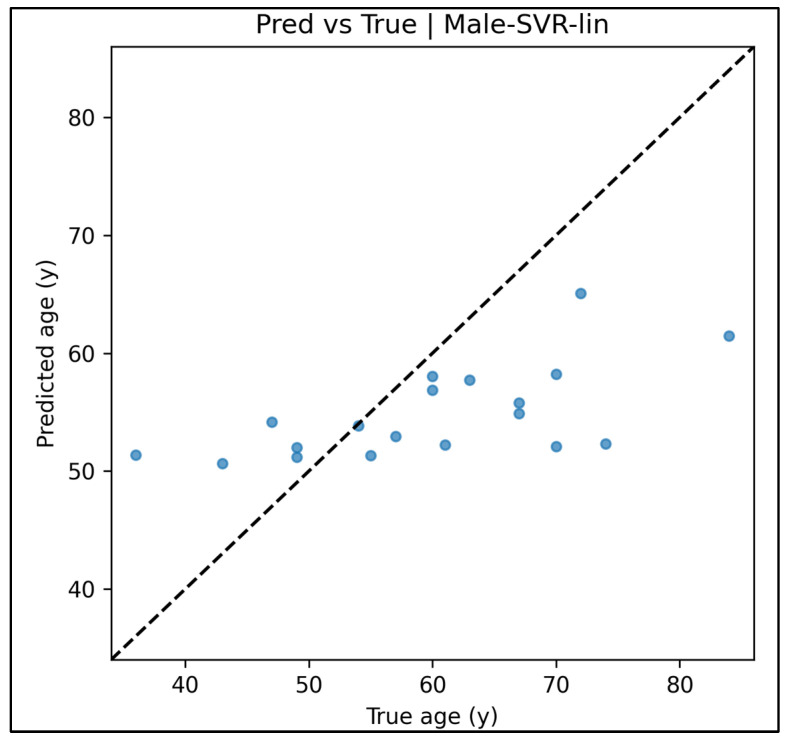
Predicted vs. true scatter plot for the male support vector regression (linear) model.

**Figure A22:**
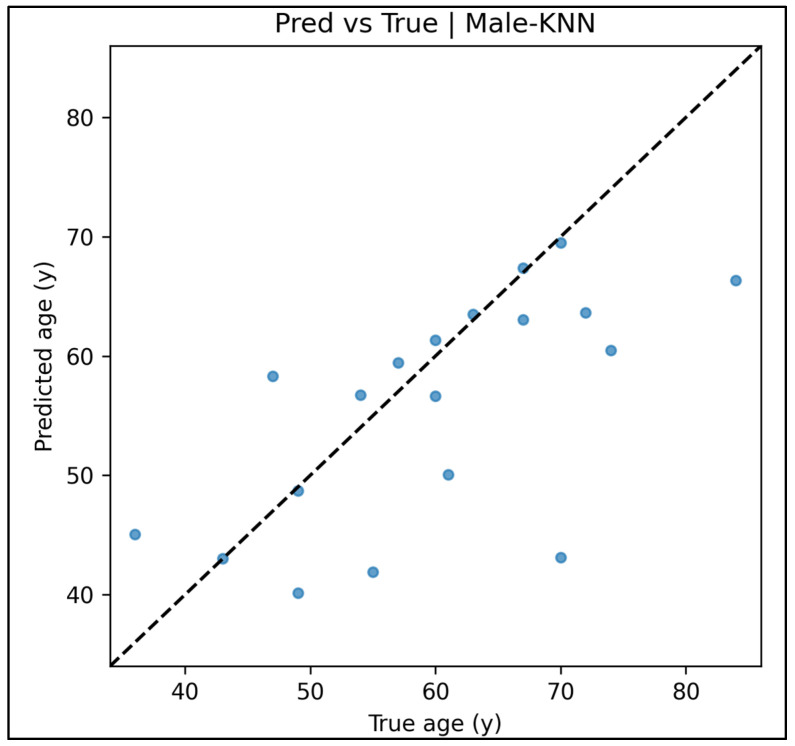
Predicted vs. true scatter plot for the male k-nearest neighbors model.

**Figure A23:**
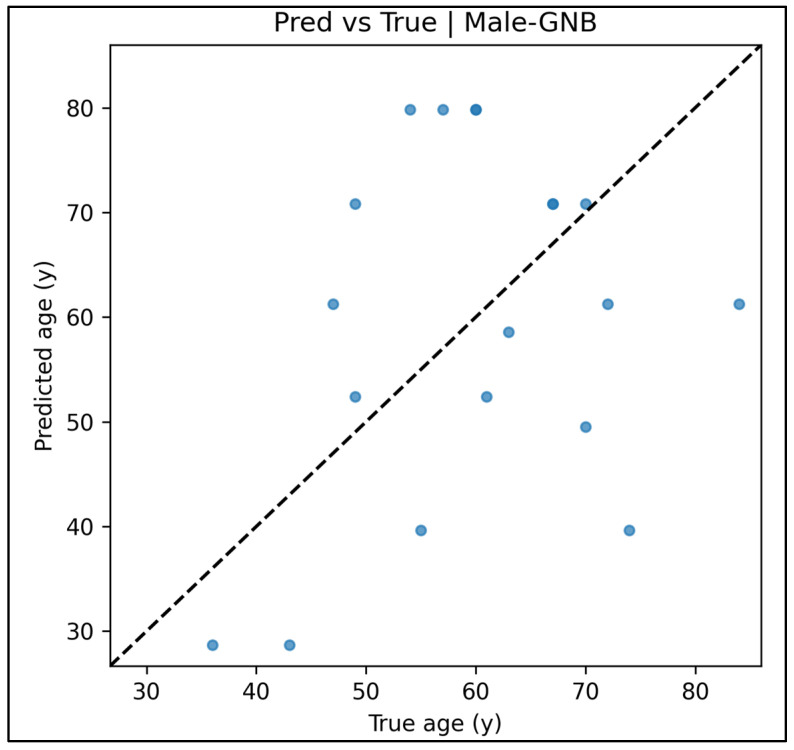
Predicted vs. true scatter plot for the male Gaussian naïve-Bayes regressor.

**Figure A24:**
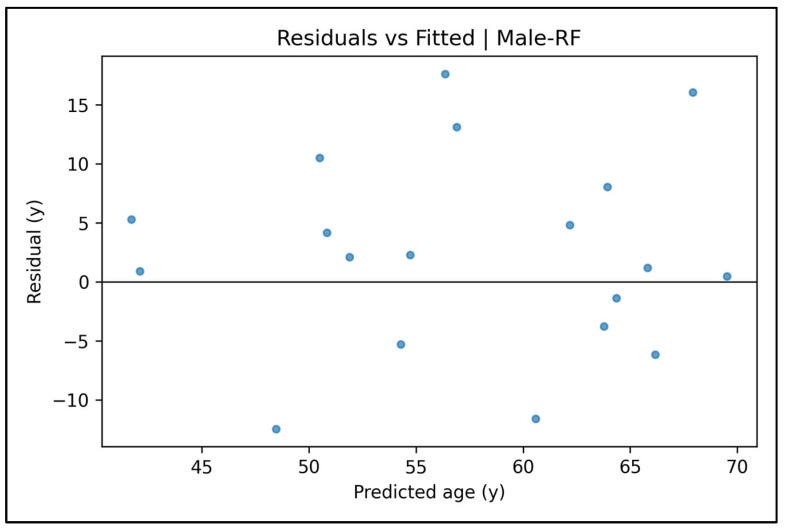
Residual vs. fitted plot for the male random forest model.

**Figure A25:**
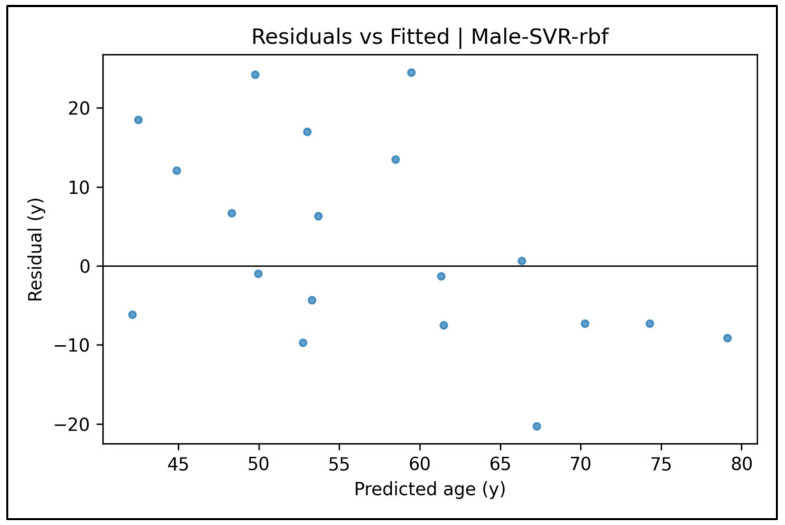
Residual vs. fitted plot for the male support vector regression (radial basis function) model.

**Figure A26:**
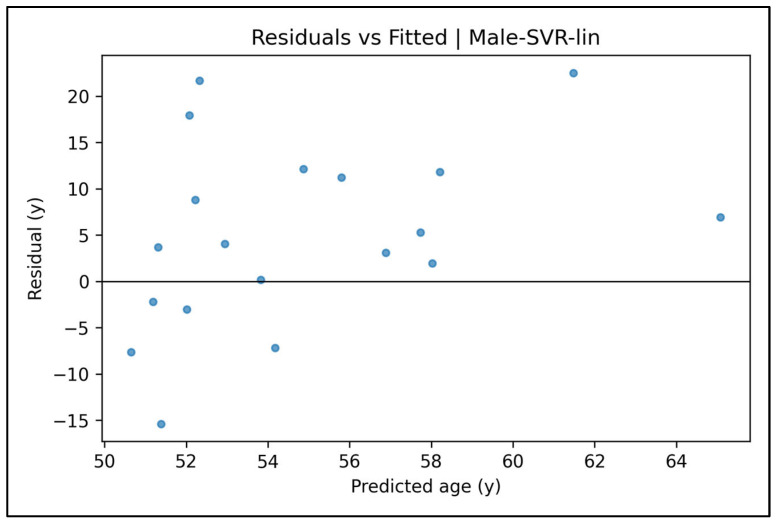
Residual vs. fitted plot for the male support vector regression (linear) model.

**Figure A27:**
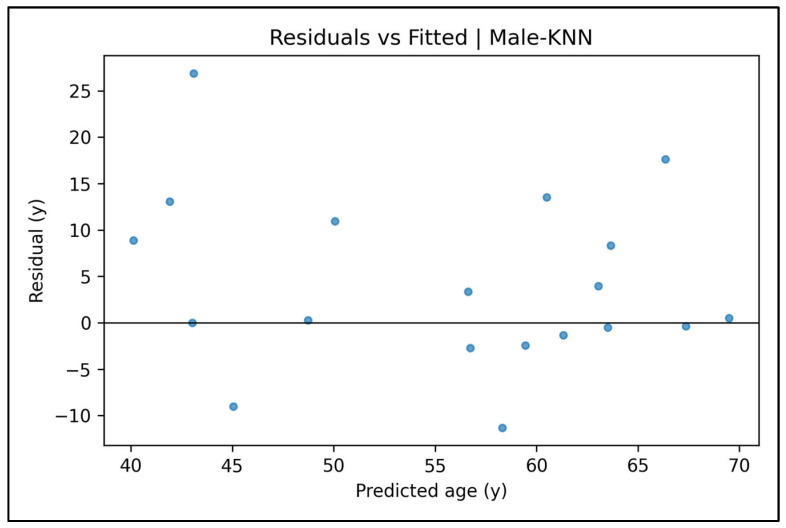
Residual vs. fitted plot for the male k-nearest neighbors model.

**Figure A28:**
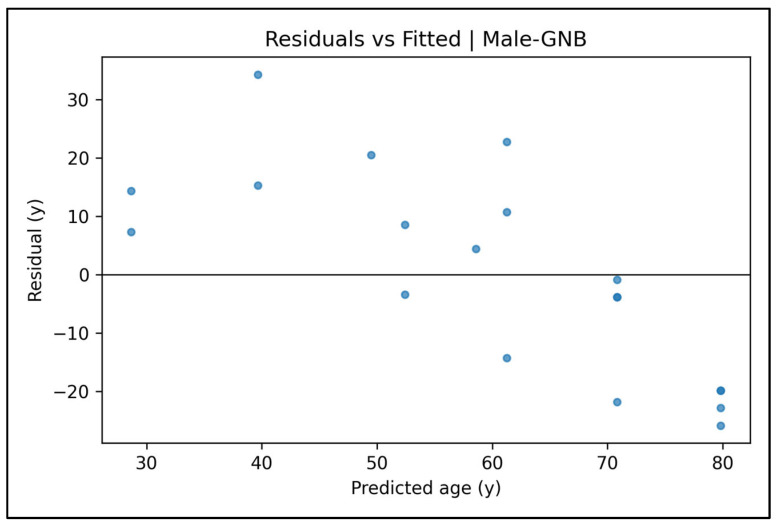
Residual vs. fitted plot for the male Gaussian naïve-Bayes regressor.

**Figure A29:**
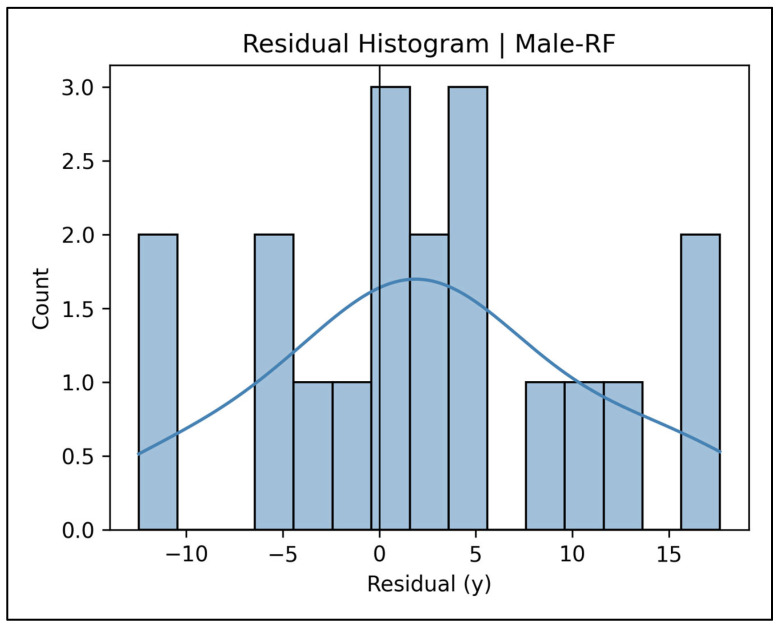
Residual histogram plot for the male random forest model.

**Figure A30:**
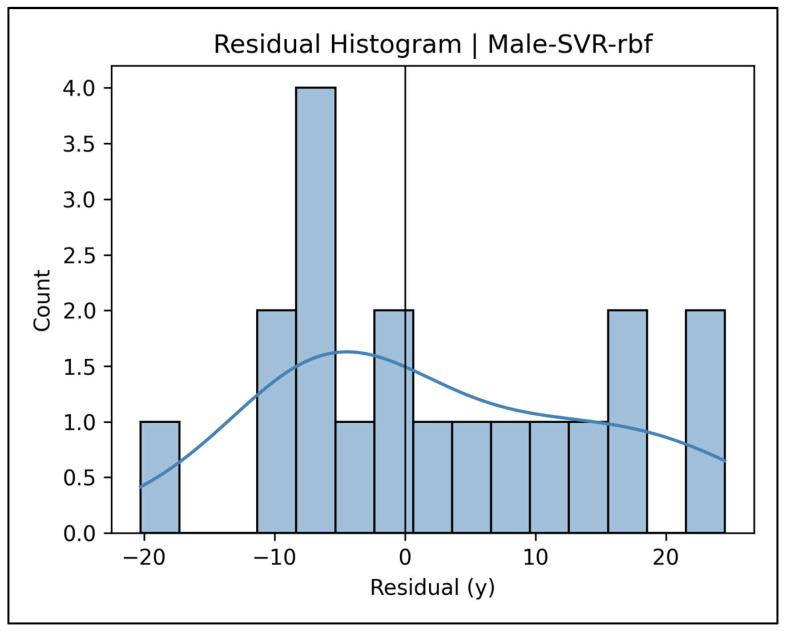
Residual histogram plot for the male support vector regression (radial basis function) model.

**Figure A31:**
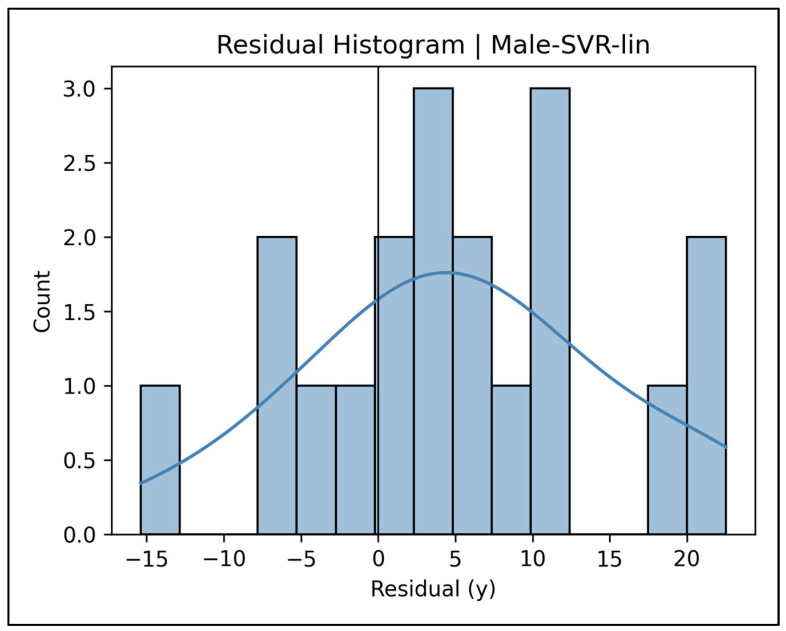
Residual histogram plot for the male support vector regression (linear) model.

**Figure A32:**
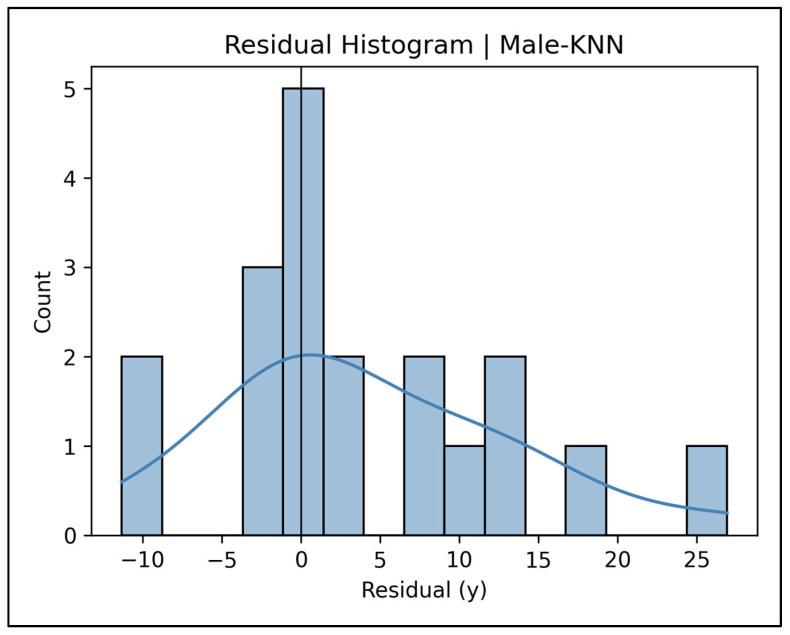
Residual histogram plot for the male k-nearest neighbors model.

**Figure A33:**
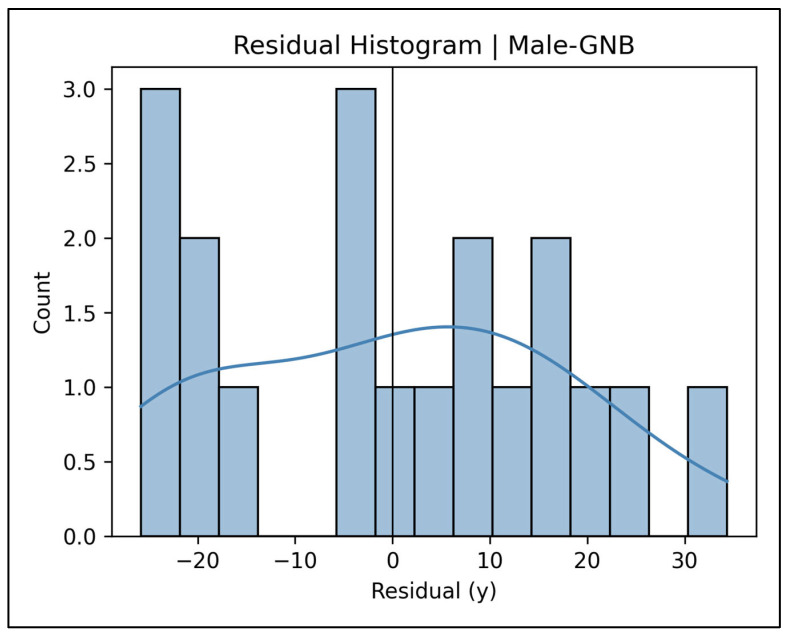
Residual histogram plot for the male Gaussian naïve-Bayes regressor.

**Figure A34:**
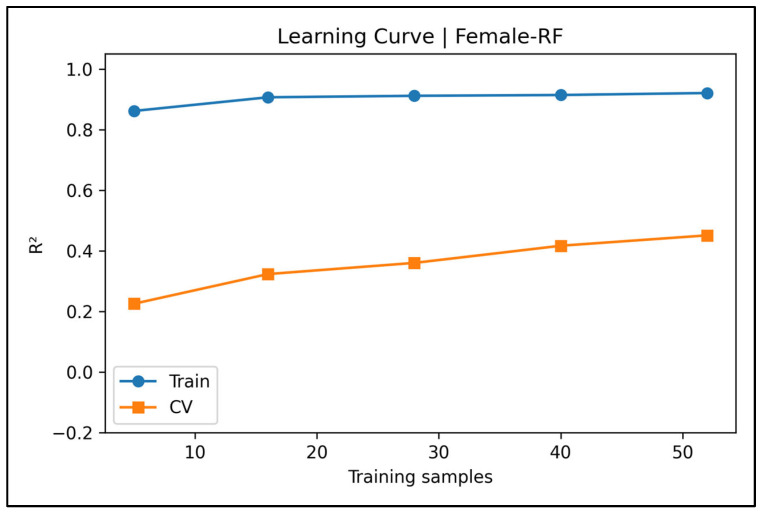
Learning curve for the female random forest model.

**Figure A35:**
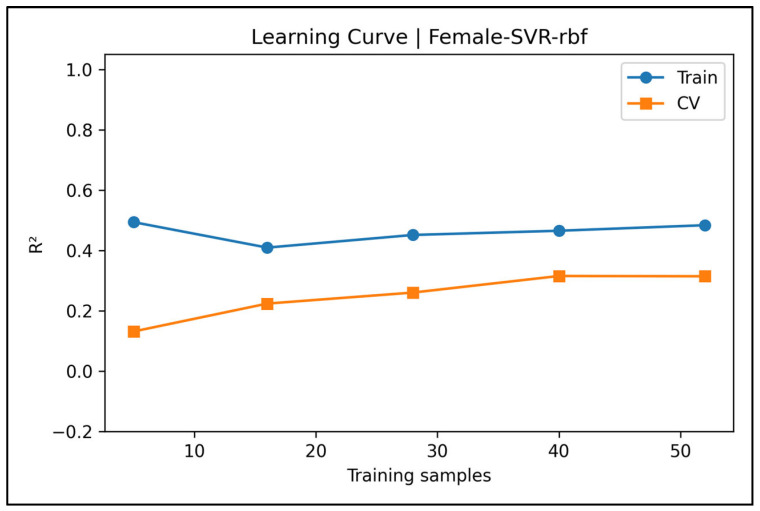
Learning curve for the female support vector regression (radial basis function) model.

**Figure A36:**
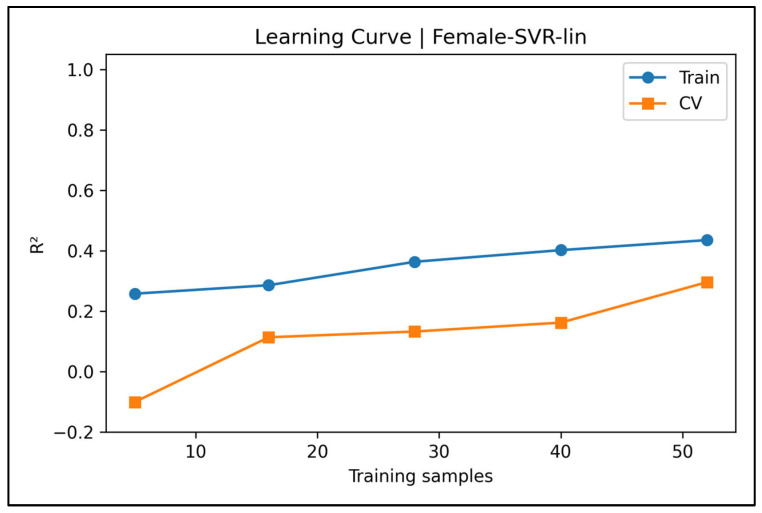
Learning curve for the female support vector regression (linear) model.

**Figure A37:**
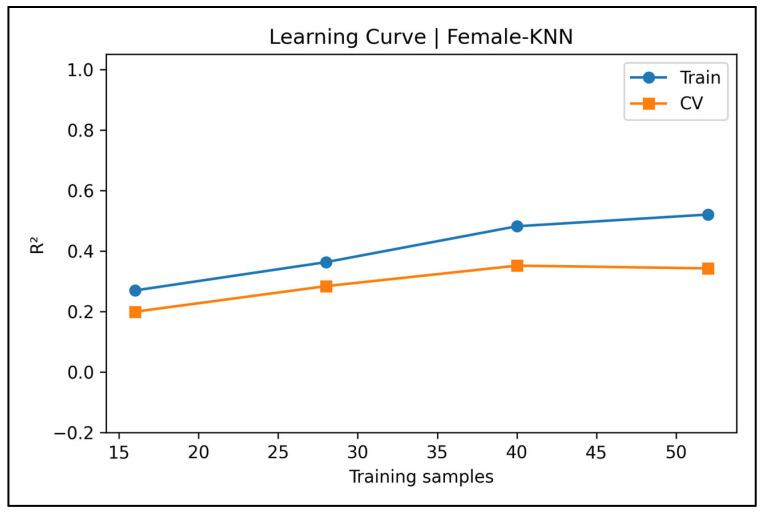
Learning curve for the female k-nearest neighbors model.

**Figure A38:**
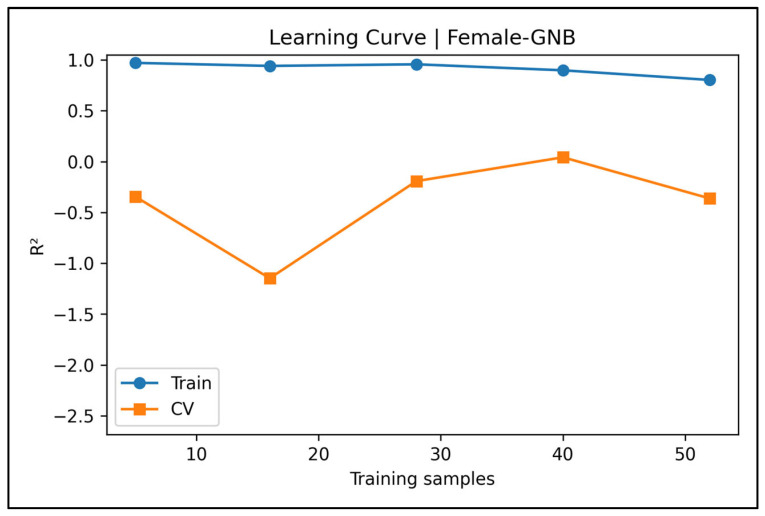
Learning curve for the female Gaussian naïve-Bayes regressor.

**Figure A39:**
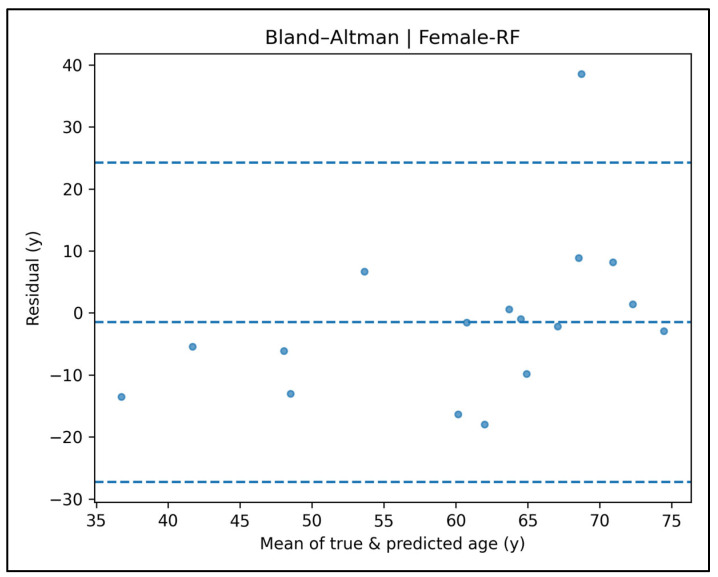
Bland–Altman plot for the female random forest model.

**Figure A40:**
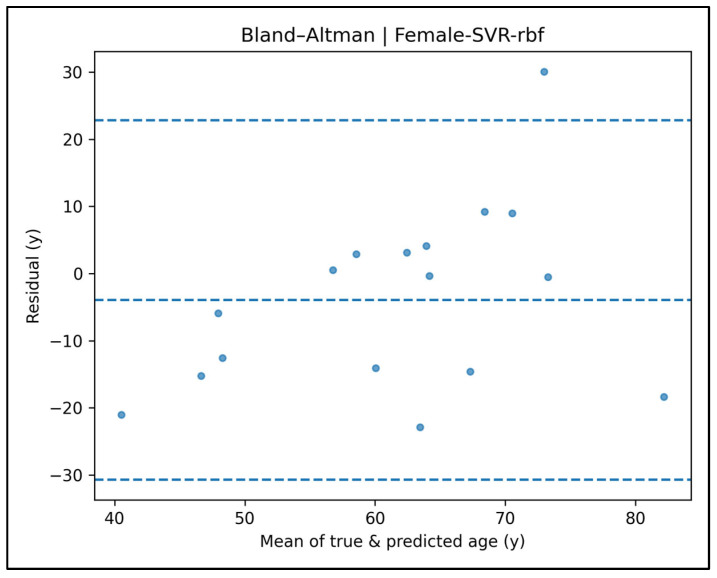
Bland–Altman plot for the female support vector regression (radial basis function) model.

**Figure A41:**
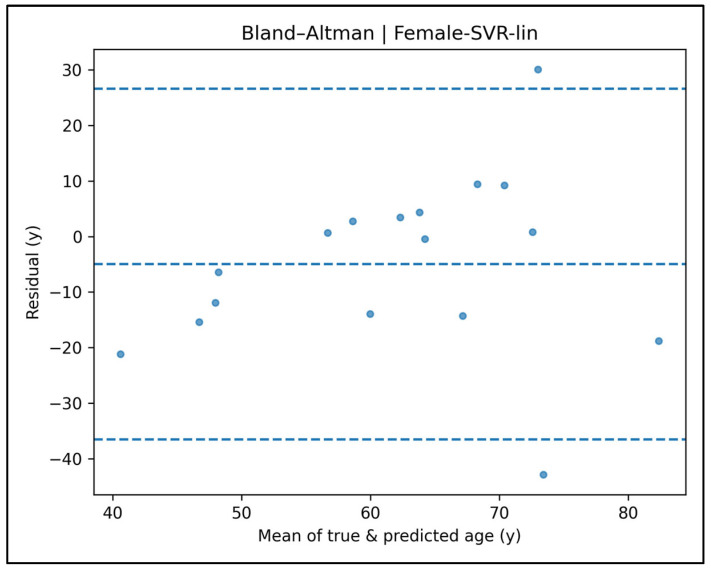
Bland–Altman plot for the female support vector regression (linear) model.

**Figure A42:**
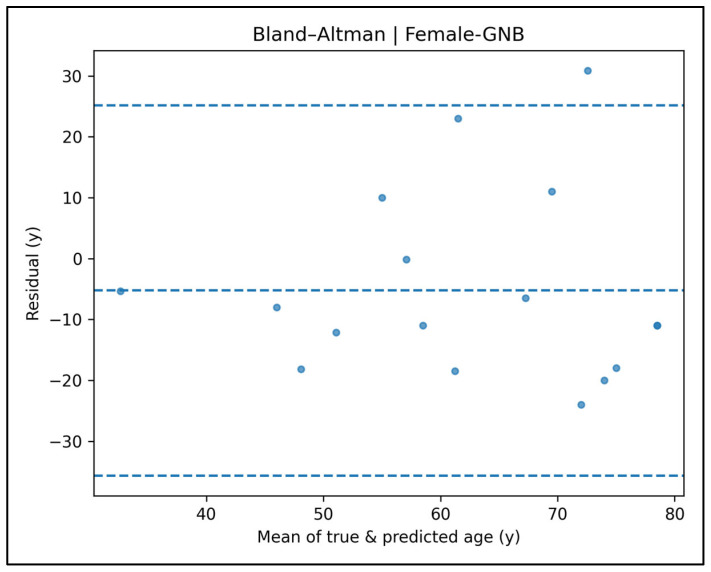
Bland–Altman plot for the female k-nearest neighbors model.

**Figure A43:**
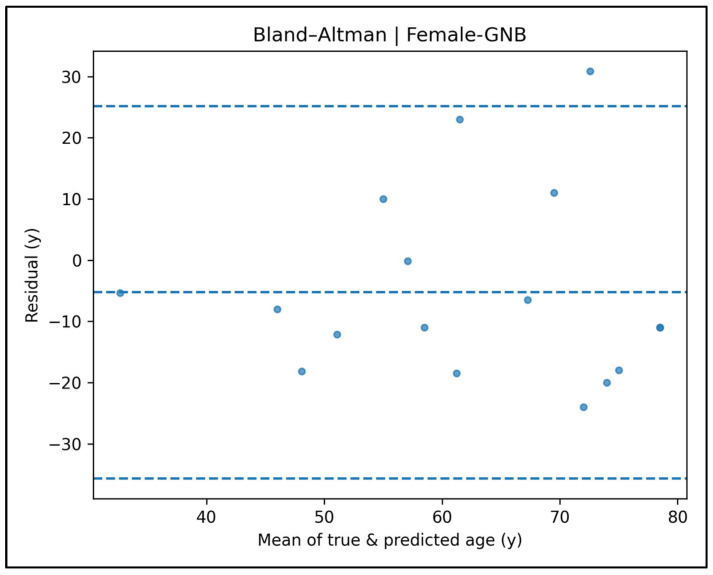
Bland–Altman plot for the female Gaussian naïve-Bayes regressor.

**Figure A44:**
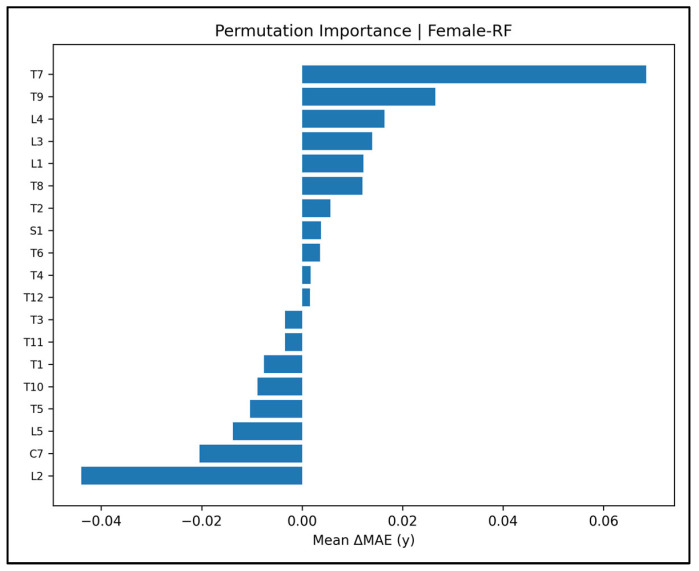
Permutation importance plot for the female random forest model.

**Figure A45:**
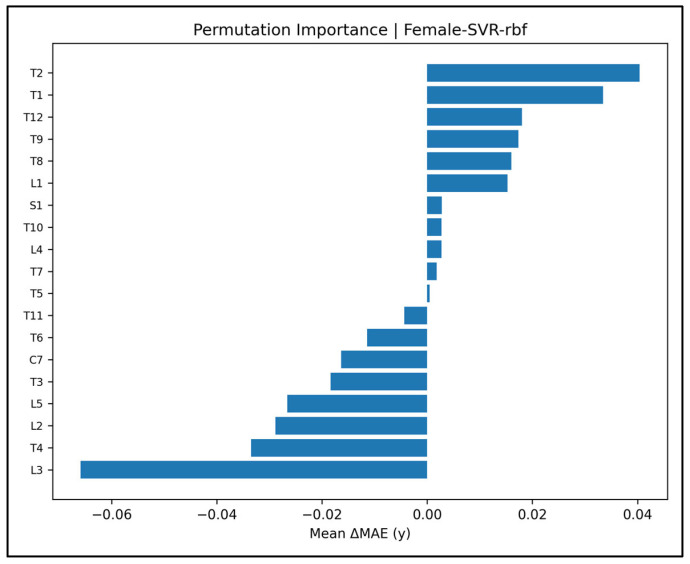
Permutation importance plot for the female support vector regression (radial basis function) model.

**Figure A46:**
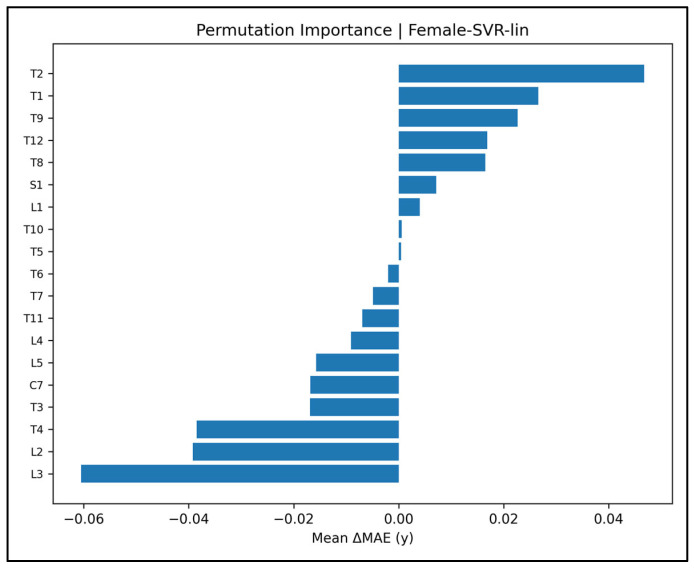
Permutation importance plot for the female support vector regression (linear) model.

**Figure A47:**
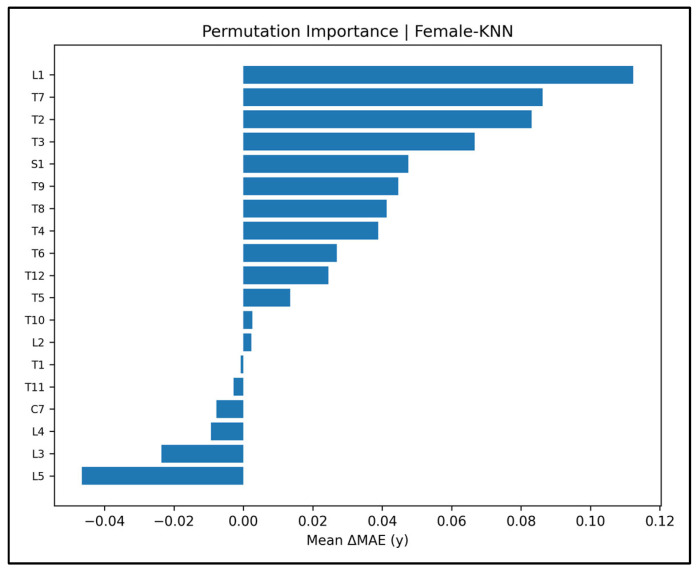
Permutation importance plot for the female k-nearest neighbors model.

**Figure A48:**
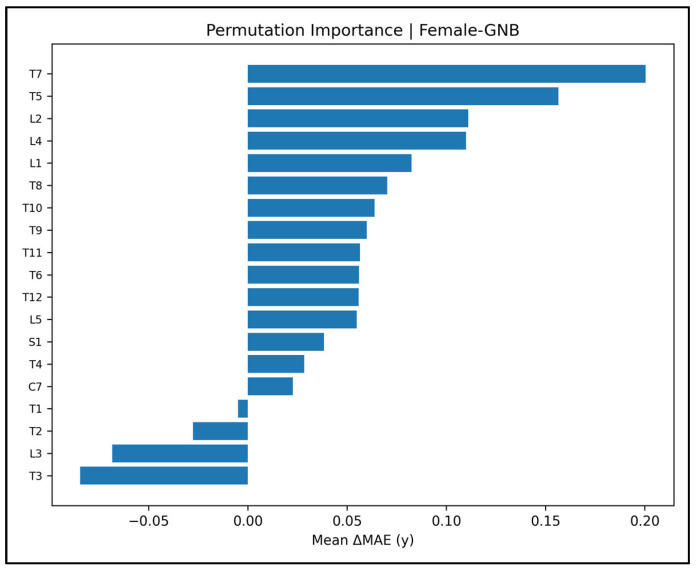
Permutation importance plot for the female Gaussian naïve-Bayes regressor.

**Figure A49:**
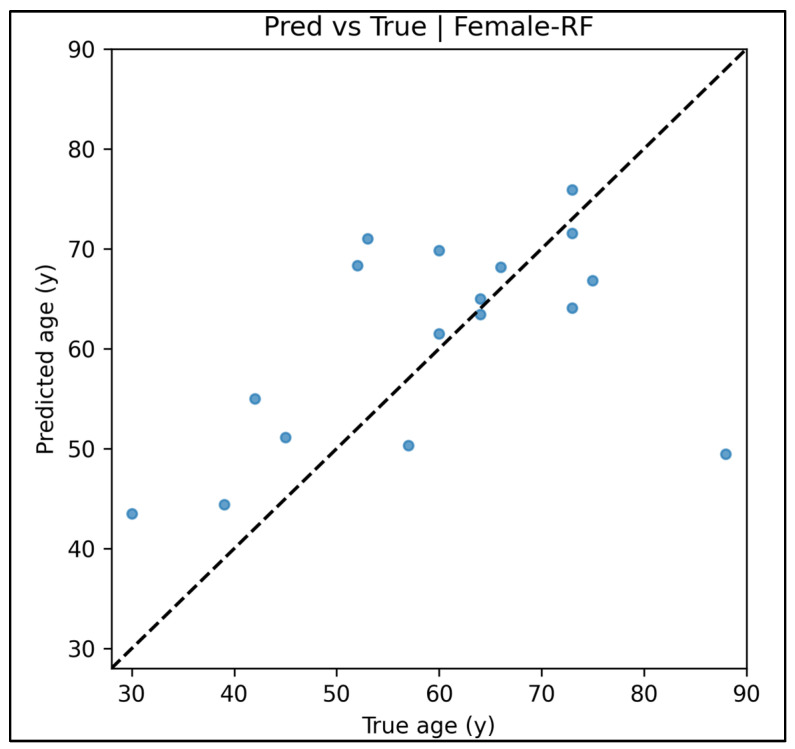
Predicted vs. true scatter plot for the female random forest model.

**Figure A50:**
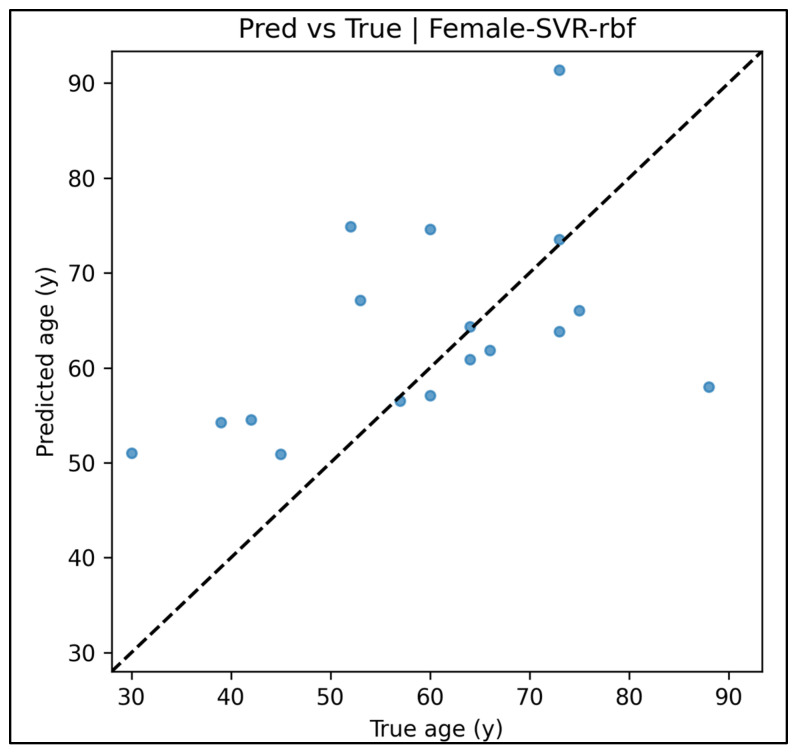
Predicted vs. true scatter plot for the female support vector regression (radial basis function) model.

**Figure A51:**
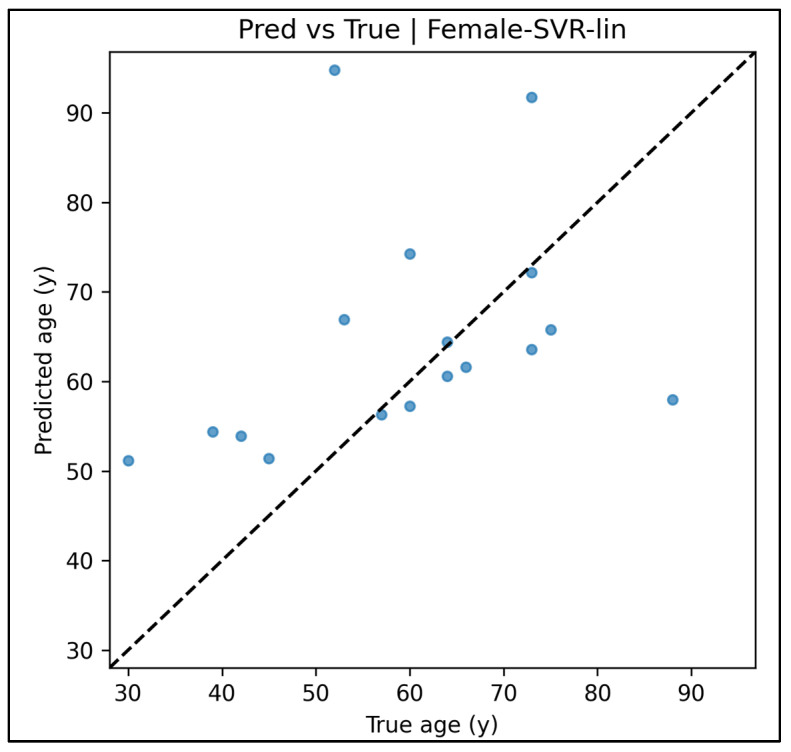
Predicted vs. true scatter plot for the female support vector regression (linear) model.

**Figure A52:**
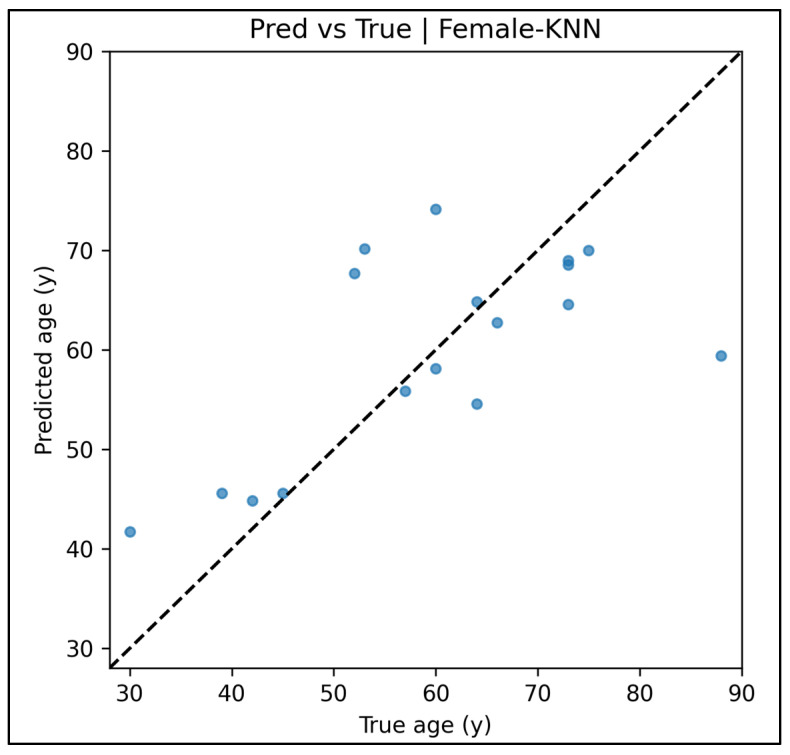
Predicted vs. true scatter plot for the female k-nearest neighbors model.

**Figure A53:**
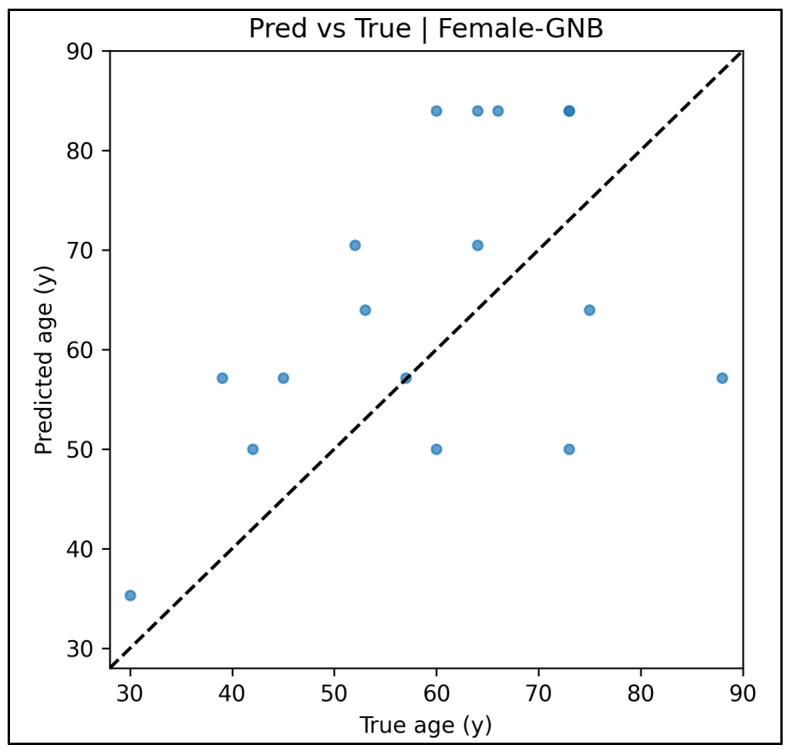
Predicted vs. true scatter plot for the female Gaussian naïve-Bayes regressor.

**Figure A54:**
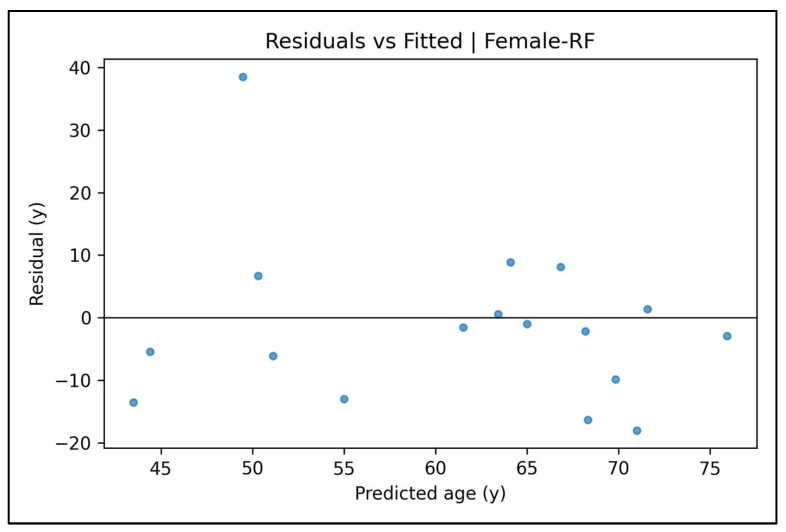
Residual vs. fitted plot for the female random forest model.

**Figure A55:**
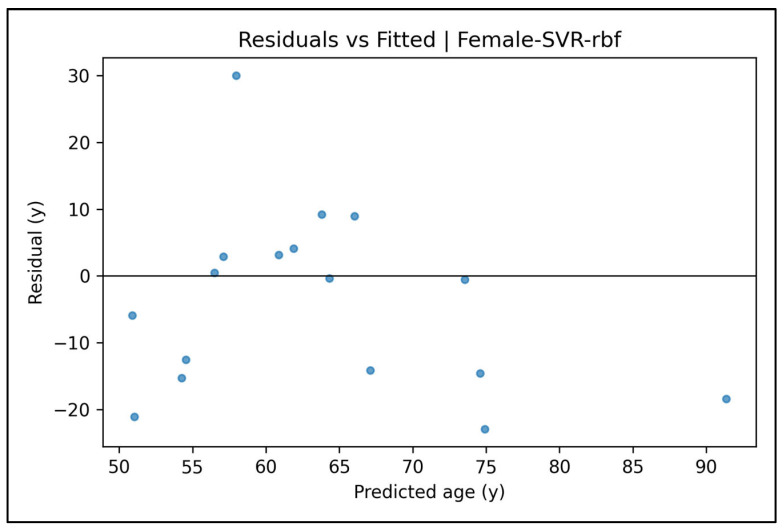
Residual vs. fitted plot for the female support vector regression (radial basis function) model.

**Figure A56:**
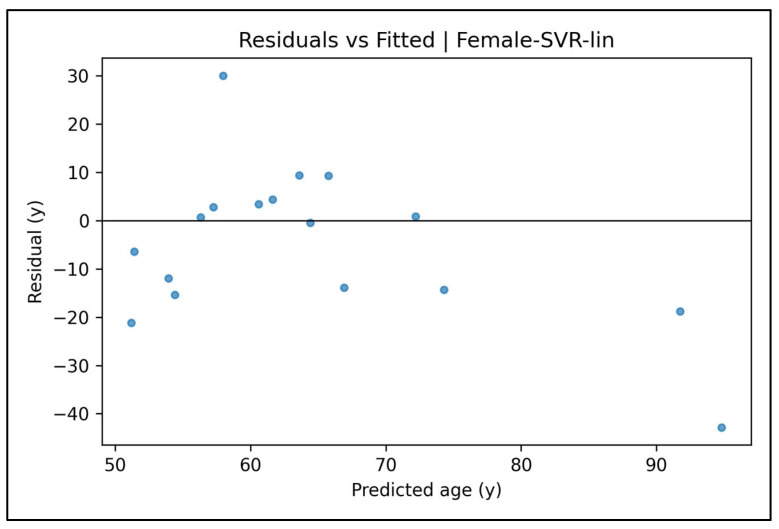
Residual vs. fitted plot for the female support vector regression (linear) model.

**Figure A57:**
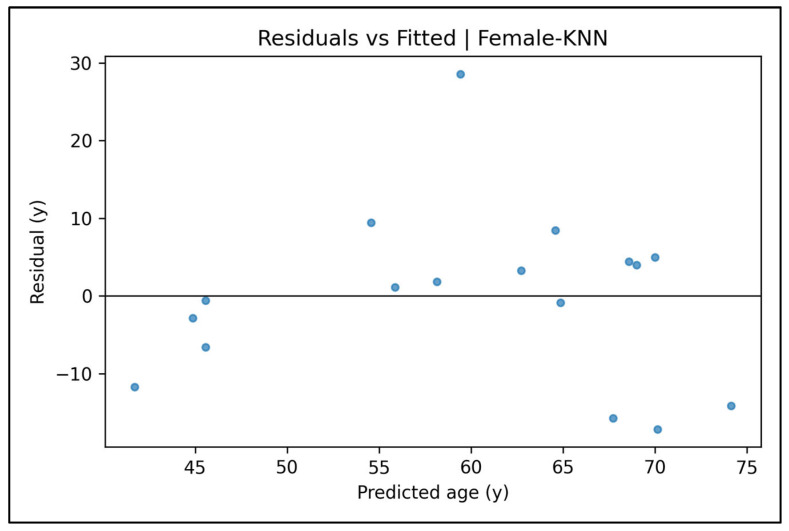
Residual vs. fitted plot for the female k-nearest neighbors model.

**Figure A58:**
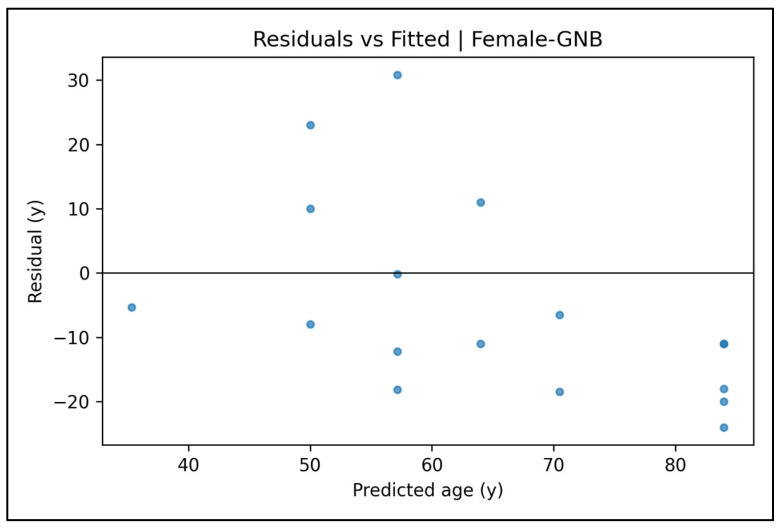
Residual vs. fitted plot for the female Gaussian naïve-Bayes regressor.

**Figure A59:**
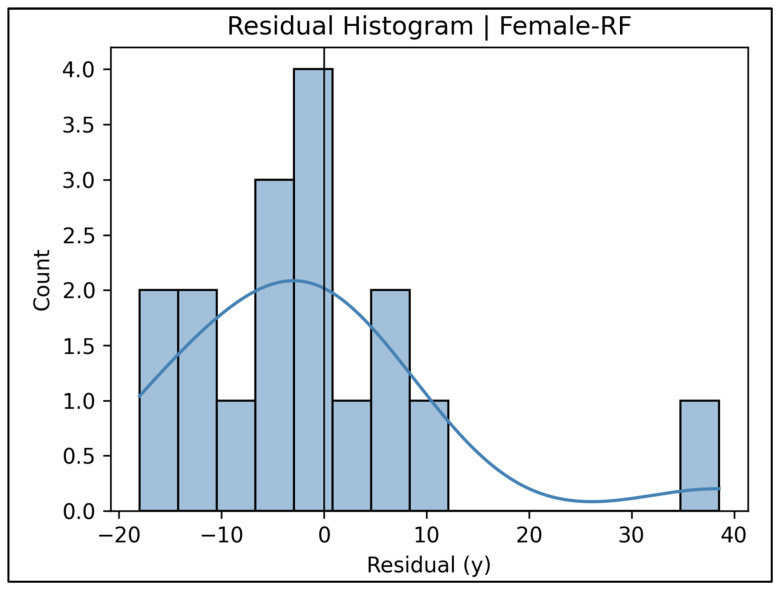
Residual histogram plot for the female random forest model.

**Figure A60:**
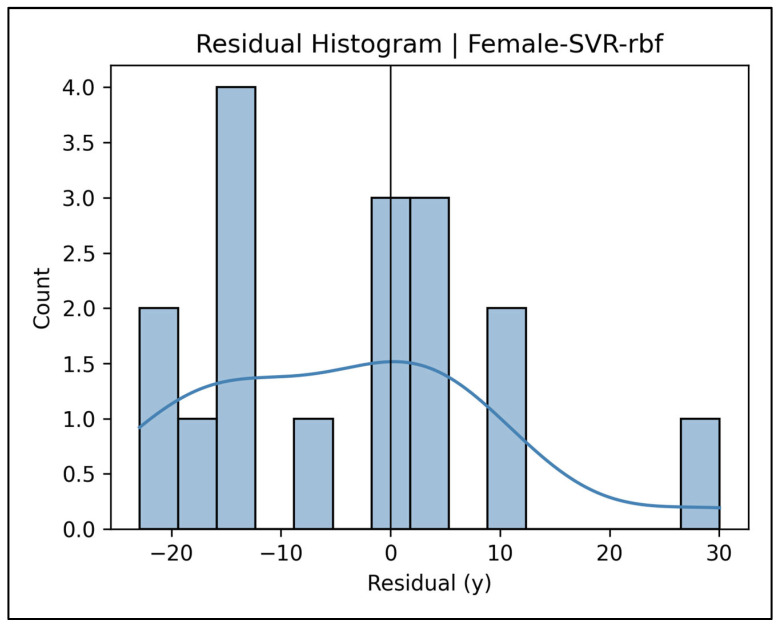
Residual histogram plot for the female support vector regression (radial basis function) model.

**Figure A61:**
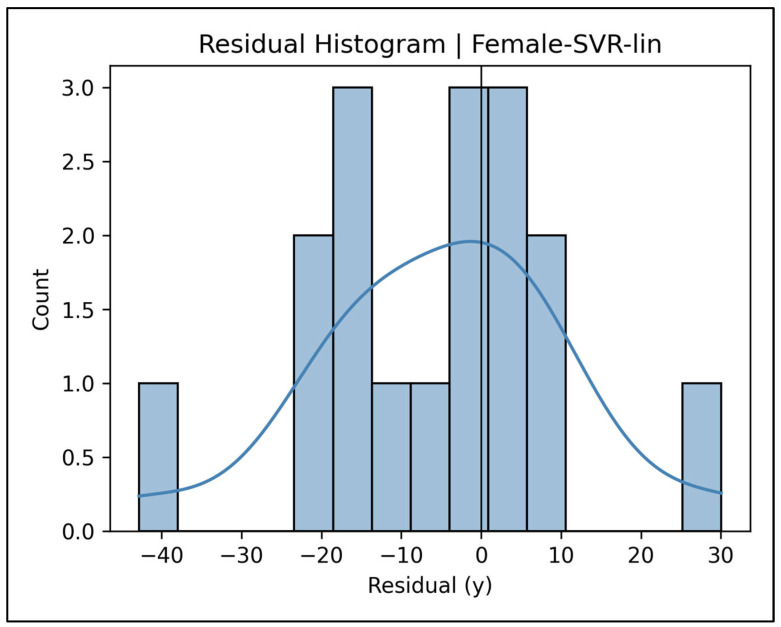
Residual histogram plot for the female support vector regression (linear) model.

**Figure A62:**
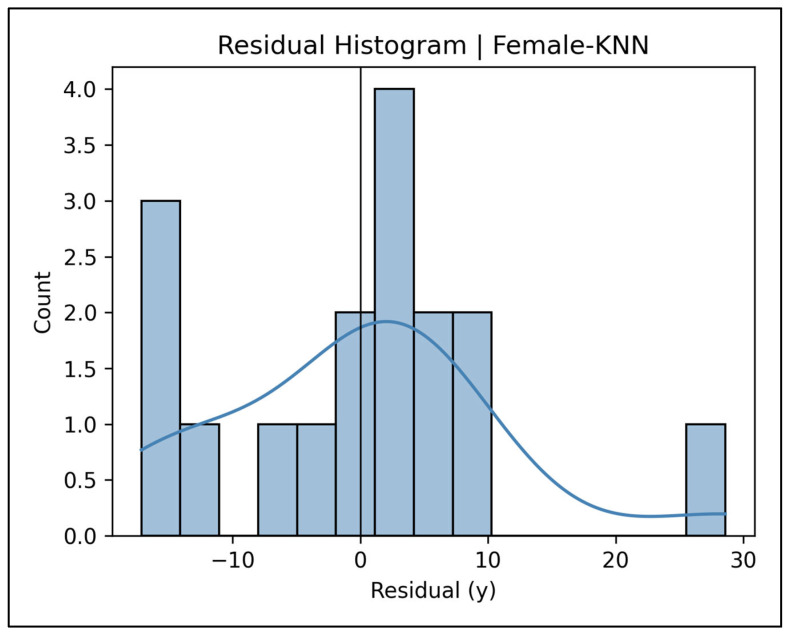
Residual histogram plot for the female k-nearest neighbors model.

**Figure A63:**
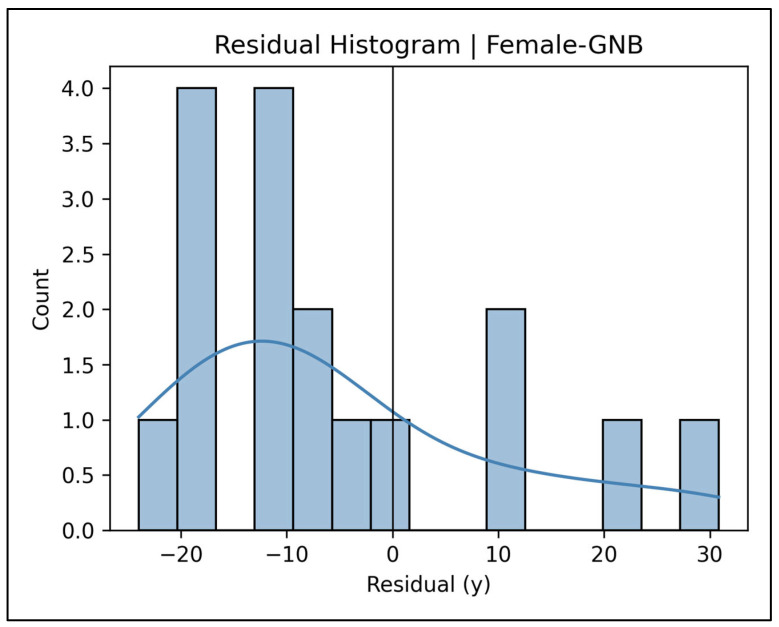
Residual histogram plot for the female Gaussian naïve-Bayes regressor.

## Data Availability

The data supporting the findings of this study are publicly available in Zenodo at https://doi.org/10.5281/zenodo.15564180. This repository includes (1) RESULTS.xlsx, containing the distance-to-ellipse (DS) scores and chronological age for each of the 176 subjects; (2) SVR_model.pkl, RF_model.pkl, GNB_model.pkl, and KNN_model.pkl, the serialized machine learning models used to generate age estimates; and (3) a README.txt describing file contents, variable definitions, and software requirements.

## Appendix A

### Appendix A.1. Age Distribution of Cases

**Table A1:** Descriptive statistics and age-group distribution of the study sample by sex. The upper panel summarizes central tendency measures (mean ± SD, median (IQR), and range) for age, while the lower panel lists absolute case counts in decadal age bins.

**Measures of Central Tendency for Age**				
	**Total Sample**	**Males**		**Females**
*N*	176	94		82
Mean ± SD	58.5 ± 14.0	57.0 ± 13.2		60.2 ± 14.7
Median (IQR)	60.0 (18.8)	58.5 (17.3)		60.0 (20.3)
Range	21–95	21–85		25–95
**Distribution of Cases According to Age Groups**				
**Age Group**	**Males**		**Females**	
21–29	3		1	
30–39	6		6	
40–49	19		10	
50–59	21		20	
60–69	30		22	
70–79	10		17	
80–89	5		4	
90–99	0		2	

*N*: number of cases; SD: standard deviation; IQR: interquartile range.

### Appendix A.2. Detailed Multiple Linear Regression Analysis Results

**Table A2:** Multiple linear regression analysis results and coefficients of DS values for male age estimation.

	Unstandardized Coefficients		Standardized Coefficients	t	Sig.	Collinearity Statistics	
	B	Std. Error	Beta			Tolerance	VIF
Constant	46.452	4.789		9.701	0.000		
C7	1.777	3.665	0.046	0.485	0.629	0.895	1.118
T1	−16.004	8.915	−0.188	−1.795	0.077	0.743	1.347
T2	−7.800	6.335	−0.133	−1.231	0.222	0.700	1.429
T3	4.118	8.767	0.051	0.47	0.640	0.698	1.432
T4	−5.765	8.731	−0.072	−0.66	0.511	0.685	1.46
T5	−0.129	8.727	−0.002	−0.015	0.988	0.553	1.809
T6	−0.829	7.127	−0.013	−0.116	0.908	0.661	1.512
T7	2.550	7.768	0.037	0.328	0.744	0.655	1.528
T8	−0.481	8.205	−0.008	−0.059	0.953	0.424	2.359
T9	9.864	9.526	0.131	1.035	0.304	0.507	1.971
T10	0.645	5.355	0.014	0.12	0.904	0.589	1.697
T11	−1.740	5.065	−0.040	−0.344	0.732	0.594	1.682
T12	0.821	4.903	0.020	0.167	0.868	0.597	1.676
L1	−1.368	6.173	−0.026	−0.222	0.825	0.577	1.732
L2	5.756	4.323	0.171	1.332	0.187	0.492	2.033
L3	13.878	4.292	0.426	3.234	0.002	0.468	2.137
L4	0.005	3.232	0.000	0.002	0.999	0.449	2.225
L5	1.429	4.940	0.039	0.289	0.773	0.450	2.223
S1	4.726	4.367	0.112	1.082	0.283	0.756	1.323

**Table A3:** Multiple linear regression analysis results and coefficients of DS values for female age estimation.

	Unstandardized Coefficients		Standardized Coefficients	t	Sig.	Collinearity Statistics	
	B	Std. Error	Beta			Tolerance	VIF
Constant	42.974	5.011		8.576	0.000		
C7 DS	−3.223	6.484	−0.071	−0.497	0.621	0.422	2.372
T1 DS	−8.138	7.034	−0.141	−1.157	0.252	0.582	1.719
T2 DS	13.016	9.327	0.178	1.396	0.168	0.532	1.879
T3 DS	−1.765	14.115	−0.018	−0.125	0.901	0.442	2.261
T4 DS	14.262	10.591	0.173	1.347	0.183	0.526	1.901
T5 DS	0.051	2.987	0.002	0.017	0.986	0.935	1.07
T6 DS	1.799	9.097	0.028	0.198	0.844	0.444	2.251
T7 DS	−9.794	9.464	−0.111	−1.035	0.305	0.753	1.328
T8 DS	15.991	10.782	0.228	1.483	0.143	0.367	2.721
T9 DS	10.704	7.059	0.185	1.516	0.135	0.582	1.718
T10 DS	0.348	9.074	0.005	0.038	0.969	0.476	2.101
T11 DS	−7.186	7.710	−0.145	−0.932	0.355	0.362	2.763
T12 DS	−3.296	3.881	−0.104	−0.849	0.399	0.576	1.735
L1 DS	5.593	14.074	0.059	0.397	0.692	0.401	2.496
L2 DS	11.203	6.644	0.282	1.686	0.097	0.311	3.216
L3 DS	1.008	1.65	0.078	0.611	0.543	0.532	1.880
L4 DS	4.870	4.314	0.153	1.129	0.263	0.475	2.107
L5 DS	3.338	4.522	0.103	0.738	0.463	0.447	2.236
S1 DS	−2.306	3.682	−0.064	−0.626	0.533	0.827	1.209

### Appendix A.3. Learning Curve Panels—Male Cases (Figure A4, Figure A5, Figure A6, Figure A7 and Figure A8)

The random forest learning curve reveals a classic variance-limited pattern: training R^2^ stabilizes around 0.90, while CV R^2^ climbs steadily from ≈ 0 to 0.40 as the training set grows, suggesting that additional cases should yield further—but diminishing—gains.

The support vector regression (radial basis function) learning curve reveals perfect memorization on the training set (train R^2^ ≈ 1.0), while CV R^2^ climbs only to ~0.40 with 60 training cases, suggesting that additional data would partly—but not fully—alleviate variance error.

The support vector regression (linear) learning curve depicts under-fitting rather than variance error: training R^2^ hovers near 0.25 and CV R^2^ stays below 0.20 across all sample sizes, suggesting that a linear decision boundary is insufficient to exploit the vertebral roughness pattern.

The k-nearest neighbors learning curve indicates perfect memorization on the training set (train R^2^ ≈ 1.0) while cross-validated R^2^ plateaus around 0.25 as sample size grows—evidence that KNN would benefit from additional cases but is fundamentally variance-constrained.

Gaussian naïve-Bayes regressor learning curve demonstrates extreme over-fitting: training R^2^ remains above 0.95 throughout, while CV R^2^ starts negative and never exceeds 0.10, indicating memorization rather than learning and explaining the collapse in external performance.

### Appendix A.4. Bland–Altman Plots—Male Cases (Figure A9, Figure A10, Figure A11, Figure A12 and Figure A13)

The random forest Bland–Altman plot shows a low mean bias (+2.5 y) and the tightest limits of agreement among male models (≈−15 y to +19 y), indicating both precision and absence of age-dependent drift.

The support vector regression (radial basis function) Bland–Altman plot shows a small positive mean bias (~+2 y) but wide limits of agreement (≈−22 y to +26 y); residual spread increases toward the extremes of the age spectrum.

The support vector regression (linear) Bland–Altman plot illustrates this bias and a mildly increasing spread with age, albeit narrower than RBF-SVR but wider than RF.

The k-nearest neighbors Bland–Altman plot shows mean bias of +2 y; limits of agreement span about −13 y to +25 y, wider than RF.

The Gaussian naïve-Bayes Bland–Altman plot shows limits of agreement spanning roughly −35 to +34 y and a negligible mean bias (−0.6 y), but the widening spread with increasing mean age signifies poor precision in older individuals.

### Appendix A.5. Permutation Importance Plots—Male Cases (Figure A14, Figure A15, Figure A16, Figure A17 and Figure A18)

The random forest permutation importance plot is sharply concentrated: L3 and L4 dominate (ΔMAE ≈ 0.20 y each), followed at a distance by T1 and T11. Most thoracic and sacral levels show ΔMAE near zero, underscoring the primacy of mid-lumbar roughness already highlighted by LASSO and univariate tests.

The support vector regression (radial basis function) permutation importance highlights the upper-lumbar DS scores (L3 > L2 > L4) as key drivers (ΔMAE up to 0.34 y); thoracic levels contribute modestly, while T12 shows a negative ΔMAE, implying that shuffling this feature unexpectedly reduces error—a sign of feature interaction rather than genuine importance.

The support vector regression (linear) permutation importance ranks the same upper-lumbar levels as previous models—L3 > L2 > L4 ≈ L5 ≈ T8—yet the absolute ΔMAE values are small (<0.09 y), reflecting the overall weak signal captured by the linear hyper-plane.

The k-nearest neighbors permutation importance distributes influence across several lumbar and lower-thoracic levels, with L3, L2, and L4 topping the list (ΔMAE up to 0.20 y). No single vertebra dominates, suggesting KNN relies on a broader neighborhood pattern than RF.

Gaussian naïve-Bayes permutation importance lists all 19 vertebral levels; only the mid-lumbar DS values (L3 > T6 > S1 ≈ L4) showed a meaningful impact (ΔMAE ≈ 0.05–0.30 y), whereas most thoracic and cervical features hovered around zero, suggesting that the model in effect relies on a single dominant predictor and treats the remainder as noise.

### Appendix A.6. Predicted vs. True Scatter Plots—Male Cases (Figure A19, Figure A20, Figure A21, Figure A22 and Figure A23)

In the male sample, the random forest scatter hugs the identity line between ≈ 45 y and 70 y, with only a slight widening of error beyond 75 y. By contrast, the RBF-SVR shows a clear compression bias: ages ≤ 55 y are mostly over-estimated, and ages ≥ 65 y under-estimated, driving its negative R^2^. The linear-SVR tightens this squeeze still further, forcing nearly every prediction into a narrow 50–65 y corridor and thus under-ageing men above 70 y while over-ageing those below 50 y. The k-NN regressor falls between these extremes—closely tracking the identity line for 45–65 y but fanning out past 70 y, which explains its moderate R^2^. Finally, the discretized Gaussian naïve-Bayes produces three coarse “age plateaus” and flips above the identity line for men under 55 y but below it for those over 70 y, a direct artefact of target discretization

### Appendix A.7. Residual vs. Fitted Plots—Male Cases (Figure A24, Figure A25, Figure A26, Figure A27 and Figure A28)

Across the male models, the residual versus fitted diagnostics tell a consistent story about bias and heteroscedasticity. The random forest and Gaussian naïve-Bayes both display a pronounced funnel: residuals start as high as +35 y at low fitted ages, cross zero around 60 y, and plunge to −20/−25 y thereafter—textbook evidence of widening variance with age. A similar, though shallower, downward slope is seen in the RBF-SVR (from +24 y to −20 y), again signaling age-dependent heteroscedasticity. The linear-SVR shows a milder pattern, retaining a predominantly positive bias (up to +22 y) with only a few negative points, consistent with its Bland–Altman mean of about +5 y. In contrast, the k-NN residual cloud is nearly symmetric (≈−12 y to +27 y) and trend-free, indicating little systematic age bias even though variance still grows slightly at the extremes.

### Appendix A.8. Residual Histograms—Male Cases (Figure A29, Figure A30, Figure A31, Figure A32 and Figure A33)

Residual histograms reinforce the same performance hierarchy. Random forest and k-NN both approximate normality, each peaking near +2 y; random forest’s tails are nearly symmetrical (−12 y to +17 y), whereas KNN shows a modest rightward stretch that pushes the upper Bland–Altman limit to about +25 y. In stark contrast, the RBF-SVR and linear-SVR are right-skewed: the radial model centers on −6 y with a long +24 y tail (matching its larger SEE of 12.7 y), while the linear variant centers on +3 y, spans −15 y to +22 y, and yields limits of roughly −15 y to +25 y. The Gaussian naïve-Bayes histogram is outright bimodal with extended tails, violating normality and explaining its very large SEE—further evidence of the model’s poor fit.

### Appendix A.9. Learning Curve Panels—Female Cases (Figure A34, Figure A35, Figure A36, Figure A37 and Figure A38)

Random forest training R^2^ is already high with six cases (≈0.86) and plateaus at ≈0.92, while the cross-validation (CV) trajectory rises steadily from ≈0.22 to ≈0.45 as the sample grows. The persistent gap of ≈0.45 R^2^ units signals a variance-limited learner: the forest extracts the underlying pattern early, does not over-fit further, and would mainly profit from additional data to tighten its generalization band.

Support vector regression (radial basis function) training R^2^ begins around 0.50 with six cases, dips to ≈0.40 when the sample reaches twenty, and then climbs back to ≈0.48 at fifty-two cases. The cross-validation (CV) trajectory rises in parallel from ≈0.15 to ≈0.30, maintaining a fairly constant gap of ≈0.15–0.20. This pattern signals a bias-limited learner that is not grossly over-fitting yet still under-utilizes the available nonlinear information; additional data should therefore translate into incremental gains rather than expose new instability.

Support vector regression (linear) training R^2^ creeps from ≈0.25 to ≈0.43 as the sample grows, while the cross-validation (CV) trajectory rises in tandem from −0.10 to ≈0.30, maintaining a fairly constant gap of ≈0.10–0.15. The parallel curves point to a bias-limited learner: the model is not over-fitting, but its linear decision surface fails to extract the nonlinear ageing signal, so further cases alone will yield only incremental gains.

The k-nearest neighbors training R^2^ rises steadily from ≈0.27 to ≈0.50 as the sample grows, while the cross-validation (CV) curve climbs in parallel from ≈0.20 to ≈0.35 and never diverges. The roughly constant gap (≈0.10–0.15) denotes a variance-limited model: it is learning from additional cases rather than over-fitting, yet more data would still be needed to push CV R^2^ beyond the mid-0.30s.

Gaussian naïve-Bayes regressor training R^2^ started very high (≈0.9) but dropped steadily to ≈0.6 as more cases were added, while the cross-validation curve remained at or below zero throughout. The diverging trajectories corroborate severe over-fitting: additional data reduced training accuracy without yielding a concomitant gain in validation performance.

### Appendix A.10. Bland–Altman Plots—Female Cases (Figure A39, Figure A40, Figure A41, Figure A42 and Figure A43)

Random forest mean bias is negligible (–1.3 y); the 95% limits of agreement span roughly −28 y to +25 y. Omitting the +38 y outlier would contract the upper limit to ≈+21 y, underscoring the model’s generally homoscedastic behavior across the 35–80 y range.

Support vector regression (radial basis function) mean bias is about −5 y (SVR tends to under-age older women), and the 95% limits of agreement span roughly −31 y to +23 y. Although narrower than the naïve-Bayes limits (≈±30 y), the agreement band remains wider than for the female KNN (≈±25 y) and substantially broader than the best male models (RF ≈ ±15 y). The scatter is homoscedastic below ≈70 y but widens thereafter, reinforcing the observation that error dispersion increases with chronological age.

Support vector regression (linear) mean bias is around −6 y (the SVR tends to under-estimate), and the 95% limits of agreement stretch from roughly −38 y to +27 y—the widest of all female models reviewed and even broader than the RBF-SVR band. Scatter widens with the mean age, reinforcing the observation that precision deteriorates in older decades.

The k-nearest neighbors mean bias is small (+2.1 y), and the 95% limits of agreement span −22 y to +29 y—much narrower than GNB (≈±30 y) and comparable to the male KNN limits, but still wider than ensemble methods (see RF). The spread is roughly homoscedastic; only one extreme outlier (+29 y) inflates the upper limit.

Gaussian naïve-Bayes mean bias is ≈−5 y (the model tends to over-age women on average), and the 95% limits of agreement fan out from roughly −35 y to +26 y—by far the widest band in the female cohort. The point-cloud broadens as mean age rises, underscoring marked heteroscedasticity and mirroring the model’s large SEE and poor overall fit.

### Appendix A.11. Permutation-Importance Plots—Female Cases (Figure A44, Figure A45, Figure A46, Figure A47 and Figure A48)

Unlike the male forest—which locked onto L3/L4—the female ensemble focuses on the upper-thoracic to upper-lumbar arc: T7 dominates (ΔMAE ≈ 0.07 y), followed by T9 and lumbar facets L4, L3, and L1. Permuting L2 markedly improves accuracy (ΔMAE ≈ −0.04 y), implying noise or an interaction that the forest cannot reconcile. The broader, gently sloping profile (many levels with small ΔMAE) mirrors the diffuse ageing signal seen in women and explains the forest’s ability to smooth over local irregularities that confound SVR.

The support vector regression (radial basis function) model concentrates on upper thoracic facets (T2–T1) and the thoraco-lumbar junction (T12–T9, L1). Permuting lower-lumbar roughness (L3–L5) improves accuracy (negative ΔMAE), suggesting that these levels introduce noise rather than signal—a finding that mirrors the male RBF-SVR and underscores the vertex-based sensitivity of support vector machines.

The support vector regression (linear) relies almost exclusively on upper-thoracic roughness (T2 > T1 > T9 ≈ T12 ≈ T8), each contributing ΔMAE ≤ 0.05 y. Permuting mid- and lower-lumbar levels (L3 → L5) actually improves accuracy (negative bars), implying that these features inject noise rather than signal—consistent with the model’s inability to handle curvature in that region.

In k-nearest neighbors, permutation importance analysis influence is distributed across upper-lumbar (L1) and mid-thoracic (T7, T2–T3) roughness, each ΔMAE ≤ 0.11 y. Negative bars for L5–L3 suggest weak, possibly noisy contributions from the low-lumbar region. The levelled profile corroborates the neighborhood-based nature of KNN: no single vertebra dominates, unlike the RF or SVR models that lock onto L3/L4.

Although the ranking is unstable (many bars cross zero), the Gaussian naïve-Bayes regressor algorithm placed most weight on mid-thoracic (T7–T5) and upper-lumbar (L2–L4) roughness, echoing the thoraco-lumbar distribution found by the more successful female models. Several inferior thoracic levels (T3, L3) displayed negative ΔMAE, suggesting that permuting these features occasionally improved accuracy—a hallmark of noise rather than signal.

### Appendix A.12. Predicted vs. True Scatter Plots—Female Cases (Figure A49, Figure A50, Figure A51, Figure A52 and Figure A53)

In the female cohort, the random forest predictions track the identity line from ≈ 45 y to 75 y; however, random forest under-ages the oldest woman (true 88 y → pred. 50 y; +38 y) and slightly over-ages several cases in their mid-60s to early-70s (residuals ≈ −15 y). Apart from that single outlier, residuals are quasi-normal (median ≈ −1 y, IQR ±7 y) and stay within ±18 y, with no discernible slope in the residual vs. fitted plot. RBF-SVR compresses the age range to 55–75 y, over-ages the youngest decade (30–45 y), and markedly under-ages the oldest subject (true ≈ 90 y). Residuals cluster around −5 y with a shallow right tail to +30 y; the residual vs. fitted display fans out above ≈ 70 y, and the histogram is bimodal (modes ≈ −15 y and +5 y). Linear-SVR narrows predictions still further (50–75 y), systematically over-aging women under ≈ 45 y and under-aging the 90-year-old (pred. ≈ 58 y); its skew-normal histogram peaks just below zero and carries heavy tails (−42 y to +30 y), while a broad fan-shaped residual plot attests to pronounced heteroscedasticity. By contrast, k-NN adheres closely to the identity line between 50 y and 75 y. It under-estimates the lone 30-year-old and flattens slightly above 80 y, but residuals remain centered near +2 y (IQR ±8 y) with no systematic trend, and the histogram shows a compact core around +2 y with a thinner right tail to +29 y. Finally, Gaussian naïve-Bayes collapses predictions into a 55–85 y band, yielding large negative errors for women < 45 y and positive errors in the oldest decade; its bimodal histogram sits well below zero and Bland–Altman limits of −27 y to +26 y underscore the model’s limited forensic utility.

### Appendix A.13. Residual vs. Fitted Plots—Female Cases (Figure A54, Figure A55, Figure A56, Figure A57 and Figure A58)

Across the female models, residual patterns separate the better-behaved learners from the more biased ones. Gaussian naïve-Bayes and both support vector regressors trace a conspicuous downward slope: residuals are strongly positive at lower fitted ages (up to +30 y around 55–60 y) but swing increasingly negative beyond ≈75 y (reaching −20 to −40 y), producing a wide “funnel” that flags age-dependent heteroscedasticity. The slope is steepest in the linear SVR, which also shows the largest negative extreme (−42 y), underscoring its tendency to over-age the young and under-age the old. RBF-SVR follows the same trend but with a slightly narrower spread. By contrast, the non-parametric k-NN cloud is almost trend-free: residuals hover symmetrically around zero (≈−17 to +29 y) with no systematic drift, apart from a single +29 y high error at a fitted age of ≈60 y. Random forest behaves similarly, maintaining residuals within ±18 y and exhibiting no discernible slope, save for one +38 y outlier at a fitted age of ≈50 y. Taken together, these diagnostics confirm that k-NN and random forest keep age-related bias in check, whereas GNB and both SVR variants suffer marked heteroscedasticity—most acutely in the linear kernel implementation.

### Appendix A.14. Residual Histograms—Female Cases (Figure A59, Figure A60, Figure A61, Figure A62 and Figure A63)

The distribution of errors corroborates the ranking suggested by other diagnostics. Random forest shows the healthiest shape: a quasi-normal core centered just below zero, with most residuals constrained to ±18 y and only a single +40 y outlier pulling the right tail—hence the narrowest SEE among the parametric learners. The k-nearest neighbors model is similarly well behaved but marginally broader: its mode sits near +2 y and, although the left tail fades around −14 y, a thin right tail extends to +29 y, inflating the upper Bland–Altman bound. Both support vector regressors are noticeably skewed. The radial kernel SVR clusters around −6 y but trails off to +30 y, producing the long positive tail that accompanies its higher SEE (≈13.8 y). The linear kernel SVR is even more dispersed—its peak lies just below zero, but heavy bilateral tails stretch from −42 y to +30 y, making it the least Gaussian and most error-prone of the five algorithms. Finally, Gaussian naïve-Bayes deviates the most from normality: the histogram is clearly bimodal with a dominant negative mode (≈−13 y) and a sparse positive lobe reaching +30 y, mirroring the model’s systematic under-ageing of young women and over-ageing of older ones. Collectively, these histograms underline that random forest and k-NN keep residual variance in check, whereas both SVR variants and especially GNB suffer from pronounced asymmetry or multimodality that widens their prediction limits.

### Appendix A.15. Pooled Total Sample Results

**Table A4:** Multiple linear regression of average distance to the fitted ellipse scores (DS) on chronological age for pooled total sample.

Statistic	Pooled Total Sample (*n* = 176)
R	0.566
R^2^	0.321
Adjusted R^2^	0.238
SEE (years)	12.18
F (df)	3.88
Model *p*	0.000
Max VIF (variable)	2.075 (L2 DS)
Min tolerance (variable)	0.482 (L2 DS)
Max condition index	15.3
Collinearity flag	none

**Table A5:** Multiple linear regression analysis results and coefficients of DS values for pooled total sample age estimation.

	Unstandardized Coefficients		Standardized Coefficients	t	Sig.	Collinearity Statistics	
	B	Std. Error	Beta			Tolerance	VIF
Constant	43.363	3.290		13.18	0.000		
C7	3.605	3.083	0.088	1.169	0.244	0.768	1.302
T1	−12.051	4.864	−0.190	−2.477	0.014	0.741	1.350
T2	1.112	4.928	0.017	0.226	0.822	0.767	1.305
T3	4.441	6.912	0.050	0.643	0.521	0.724	1.382
T4	0.917	6.331	0.011	0.145	0.885	0.692	1.444
T5	0.365	2.747	0.009	0.133	0.894	0.927	1.079
T6	0.024	5.287	0.000	0.005	0.996	0.649	1.542
T7	−2.829	5.871	−0.036	−0.482	0.631	0.772	1.295
T8	6.925	5.899	0.107	1.174	0.242	0.519	1.925
T9	13.773	5.348	0.213	2.575	0.011	0.638	1.567
T10	0.396	4.285	0.007	0.092	0.927	0.685	1.460
T11	−0.569	3.833	−0.012	−0.148	0.882	0.620	1.613
T12	−2.887	2.872	−0.081	−1.005	0.316	0.675	1.481
L1	0.040	5.467	0.001	0.007	0.994	0.594	1.685
L2	6.241	3.417	0.174	1.826	0.070	0.482	2.075
L3	1.903	1.345	0.113	1.414	0.159	0.686	1.457
L4	2.712	2.518	0.100	1.077	0.283	0.509	1.965
L5	5.463	2.658	0.180	2.056	0.041	0.566	1.768
S1	2.387	2.587	0.064	0.923	0.358	0.894	1.119

**Table A6:** Performance of LASSO and four machine learning regressors for pooled total sample age prediction.

Group	Model	Holdout R^2^	95% CI (R^2^)	SEE (y)	95% CI (SEE)	MAE (y)	RMSE (y)	CV R^2^	OOB R^2^ †
Pooled Total	LASSO	0.061	−0.32–0.25	19.61	15.9–23.2	10.72	13.07	−0.037	-
Pooled Total	RF	0.191	−0.29–0.44	18.20	14.6–21.4	10.14	12.13	0.454	0.443
Pooled Total	SVR-rbf	0.073	−0.58–0.42	19.49	14.8–23.5	10.34	12.99	0.258	-
Pooled Total	SVR-lin	0.080	−0.22–0.22	19.41	15.4–22.9	10.77	12.94	0.014	-
Pooled Total	KNN	0.153	−0.33–0.41	18.62	15.0–21.8	10.41	12.41	0.211	-
Pooled Total	GNB-Reg	−0.989	−2.61–0.20	28.54	23.6–33.5	16.30	19.02	0.068	-

R^2^ = coefficient of determination; SEE = standard error of the estimate; MAE = mean absolute error; RMSE = root mean squared error; CV R^2^ = mean 10-fold cross-validated R^2^ calculated on the 80% training split; OOB R^2^ = out-of-bag R^2^ (internal validation metric computed only for random forest); CI (95%) = two-sided percentile bootstrap confidence interval based on 1000 resamples of the external test set; RF = random forest regressor; SVR = support vector regression; KNN = k-nearest neighbors regressor; GNB-Reg = discretized Gaussian naïve-Bayes pseudo-regressor. † Out of Bag R^2^ is calculated only for the Random Forest model.
